# Precise/not precise (PNP): A Brunswikian model that uses judgment error distributions to identify cognitive processes

**DOI:** 10.3758/s13423-020-01805-9

**Published:** 2020-09-28

**Authors:** Joakim Sundh, August Collsiöö, Philip Millroth, Peter Juslin

**Affiliations:** 1 Present Address: Department of Psychology, University of Warwick, Coventry, UK; 2 Department of Psychology, Uppsala University, Uppsala, Sweden

**Keywords:** Judgment and decision making, Mathematical models, Error distributions

## Abstract

In 1956, Brunswik proposed a definition of what he called *intuitive* and *analytic* cognitive processes, not in terms of verbally specified properties, but operationally based on the observable error distributions. In the decades since, the diagnostic value of error distributions has generally been overlooked, arguably because of a long tradition to consider the error as exogenous (and irrelevant) to the process. Based on Brunswik’s ideas, we develop the precise/not precise (PNP) model, using a mixture distribution to model the proportion of error-perturbed versus error-free executions of an algorithm, to determine if Brunswik’s claims can be replicated and extended. In Experiment 1, we demonstrate that the PNP model recovers Brunswik’s distinction between perceptual and conceptual tasks. In Experiment 2, we show that also in symbolic tasks that involve no perceptual noise, the PNP model identifies both types of processes based on the error distributions. In Experiment 3, we apply the PNP model to confirm the often-assumed “quasi-rational” nature of the rule-based processes involved in multiple-cue judgment. The results demonstrate that the PNP model reliably identifies the two cognitive processes proposed by Brunswik, and often recovers the parameters of the process more effectively than a standard regression model with homogeneous Gaussian error, suggesting that the standard Gaussian assumption incorrectly specifies the error distribution in many tasks. We discuss the untapped potentials of using error distributions to identify cognitive processes and how the PNP model relates to, and can enlighten, debates on intuition and analysis in dual-systems theories.

**Electronic supplementary material:**

The online version of this article (10.3758/s13423-020-01805-9) contains supplementary material, which is available to authorized users.

More than 60 years ago, Egon Brunswik (1956) proposed definitions of, what he referred to, as *intuitive cognitive processes*, characteristic of perceptual judgments, and *analytic cognitive processes*, often observed in symbolic and conceptual tasks. Brunswik argued that intuition typically produces a Gaussian (i.e., normal) error distribution with frequent but modestly sized errors, whereas analysis produces leptokurtic error distributions with occasional but sometimes large errors. This proposal was innovative in at least two respects: First, it provided an *operational definition* of the concepts, based on empirical and observable properties of the data. Second, it focused not only on the central tendencies of the responses (means, medians), but on the shape of the *distribution of judgment errors* produced in different judgment tasks.

These ideas were confirmed by Brunswik (1956) in an experiment with a size constancy task where the participants either judged the size of an object perceptually, or were provided with the numerical information needed to calculate the size of the object. The participants in the perceptual condition were approximately correct, with a Gaussian error distribution. The participants in the conceptual condition were mostly precisely correct, with occasional large errors due to some participants relying on the wrong analytic rule. Brunswik’s ideas were later integrated into cognitive continuum theory (Hammond, Hamm, Grassia, & Pearson, 1983), although with more emphasis on the acknowledgment that most real-life tasks involve a mix of these processes. In the decades since, the notions of “intuition” and “analysis” have become closely associated with *dual-systems theories* (e.g., Evans & Stanovich, 2013; Kahneman & Frederick, 2002; Sloman, 1996), and they have therefore taken on a somewhat different meaning. For the sake of clarity, in the following we refer to the processes identified by Brunswik as “Intuition(B)” and “Analysis(B),” honoring the original context of the ideas, and in the General Discussion, we return to a discussion of the relationships between these concepts and dual-systems theories.

The idea of using the distribution of judgment errors to identify the cognitive process has been largely overlooked in psychology, and this is certainly true for the specific properties of the error distribution that were originally proposed by Brunswik (1956). One reason for this relative lack of interest is arguably a long-standing tradition to treat errors in judgment as exogenously added to—and thus essentially irrelevant to our understanding of—the process, as captured by the routine default of adding a Gaussian (normally distributed) random error to statistical and cognitive models, with little attention to exactly how noise enters into a specific process.

Of course, this is not to say that the importance of judgment errors has been entirely ignored. Research on psychophysics and scaling have integrated the role of noise in the measurement (e.g., Green & Swets, 1966; Thurstone, 1927). It has also been acknowledged that preferences can be distorted by random noise (Bhatia & Loomes, 2017), causing preference reversals (Birnbaum & Bahra, 2012), and random noise has been used to explain various biases in probability judgments (e.g., Costello & Watts, 2014; Dawes & Mulford, 1996; Erev, Wallsten, & Budescu, 1994; Hilbert, 2012). From a more applied and methodological perspective, the cost of systematic vs. random error has also been discussed in clinical versus statistical approaches to clinical judgment (Einhorn, 1986; Meehl, 1954), and in the form of a “bias-variance trade-off” in the comparison between heuristics and optimization procedures (Gigerenzer & Brighton, 2009). The idea of using error distributions to *identify cognitive processes*, as opposed to explaining behavioral effects, appears to be largely neglected in previous research, however. A very recent exception to this claim is Albrecht, Hoffmann, Pleskac, Rieskamp, and von Helversen (in press) that uses the response distributions to study the cognitive process, although in a very different way than the one proposed in this article.

The aim of this article is twofold: First, to re-introduce the highly original, operational definitions of “Intuition(B)” and “Analysis(B)” that were originally introduced by Brunswik. Second, to develop these insights into a computational model. Additionally, it is also our ambition to nurture the legacy that is implicit in Brunswik’s proposal; that random noise in judgments need not only, or primarily, be considered a nuisance in the scientific inference, but that it can be a positive means to better understand the processes. To this end, we introduce the precise/not precise (PNP) model, using a mixture distribution to distinguish between precise and nonprecise responses relative to a predefined cognitive algorithm. We first outline its mathematical details and explore its properties through model recovery. We then apply the PNP model to experimental data, to determine if Brunswik’s claims regarding Intuition(B) and Analysis(B) can be replicated and extended. In Experiment 1, we situate the PNP model in the original context of perceptual and conceptual processes, validating that the model accurately identifies the processes emphasized by Brunswik. In Experiment 2, we apply the PNP model to several conceptual tasks, showing that both types of processes can be identified on the basis of the error distributions also in purely symbolic tasks that do not emphasize sensory encoding. In Experiment 3, we use the PNP model to confirm the quasi-rational nature often assumed for the rule-based processes in multiple-cue judgment. Lastly, we discuss the implications of the PNP model, the merits of operational definitions relative to extant dual-systems theories, and the diagnostic potential of using judgment error distributions in cognitive modeling.

## Conceptualizations of Analysis(B) and Intuition(B)

Inspired by Brunswik, we define Analysis(B) to refer to deterministic (noise-free) application of explicit integration rules to exact (noise-free) and symbolic representations of cues. Due to their systematic and explicit nature, these algorithms tend to almost always produce the same result. For example, encoding that the base and the height of a right-angled triangle are 5 and 4 cm, retrieving that the area of a right-angled triangle is a product of its base and height divided by two, and retrieving the arithmetic facts that 5 × 4 = 20 and 20 / 2 = 10, will provide the same overt response (10 cm^2^). The ideal realm of Analysis(B) is error-free execution of deduction, calculus, or similar algorithms. Even though all these procedures ultimately originate in the human mind, we expect that people are, at best, able to mentally execute simple such procedures with unaided cognition, for example one-step deductions or “number crunching” of simple algebraic rules (e.g., mentally computing the area of a triangle). The errors that arise with Analysis(B) will typically involve systematic misinterpretation of the symbols, application of the wrong explicit cue integration rule (as in Brunswik’s original empiric demonstration), or erroneous execution of the integration rule. To the extent that the integration required by the algorithm is feasible to perform within the constraints of working memory, these errors can be expected to be relatively rare, but often systematic and potentially large in magnitude.

Intuition(B) algorithms depart from the generally error-free procedures of Analysis(B) in one (or both) of two ways. First, there might be error-perturbed encoding of the cues, for example due to neural noise, such as when visually assessing the distance to a place in front of oneself or when assessing the similarity between an object and a prototype (i.e., what Tversky & Kahneman, 1983, called “natural assessments”). Second, there might be an inability to consistently and reliably apply the cue integration rule in the same way on all trials, leading to different judgments from time to time for the same stimulus (cf. “lack of cognitive control” or “inconsistency”; see Brehmer, 1994; Karelaia & Hogarth, 2008). This is often observed in multiple-cue judgment where participants have rule-like beliefs about how the cues relate to a criterion (e.g., fever is a cue for pneumonia), but the cues are informally integrated rather than “number-crunched” according to a formula. With Intuition(B), judgment errors are ubiquitous, but—to the extent that the process is well tuned to the task—their magnitude is typically small. Because of the nonsymbolic nature and inherent variability of the process, it is presumably harder to succinctly summarize the process in verbal terms. Analysis(B) is thus more likely in conceptual tasks and Intuition(B) is more likely in perceptual tasks. We aim to show that, given suitable candidate algorithms, application of the PNP model, which identifies the contributions by both Analysis(B) and Intuition(B) processes, is both straightforward and reliable.

## The precise/not precise (PNP) model

We delineate two potential ways to realize a cognitive process: (i) error-free application of a cognitive algorithm, and (ii) responses that are affected by errors in the execution of the algorithm. In this context, Intuition(B) is defined by ubiquitous deviations from the algorithm, as described by the sampling from a *homogeneous* Gaussian error distribution, and Analysis(B) is defined by sampling from a *heterogeneous* error distribution, effectively sampling both error-free and error-perturbed responses. While Brunswik mainly emphasized a dichotomy between Intuition(B) and Analysis(B), cognitive continuum theory (Hammond et al., 1983) emphasizes that most real-life tasks are likely to fall on a continuum that involves a mix between these processes.

One advantage of the PNP model is that errors can be assessed in relation to any function rather than specifically in relation to the central tendency of responses or, as was often the case in previous research, the correct criterion values (Brunswik, 1956; Dunwoody, Haarbauer, Mahan, Marino, & Tang, 2000; Hammond et al., 1983; Peters, Hammond, & Summers, 1974). Therefore, we refer to responses as *precise* and *not precise,* relative to *some specific algorithm*, which may or may not be the “correct” algorithm for the task from some normative standpoint.

### Model definition

In standard cognitive modeling, a model *g*(**x**|**θ**) mapping a stimulus vector **x** and a parameter vector **θ** into predicted judgments *y* is fitted to data by least squares minimization or maximum likelihood estimation. It is typically assumed that the output of the model is perturbed by a normally (Gaussian) and independently distributed random error *N*(0, *σ*^2^) with zero expectation and standard deviation *σ*, so that

1 $$ y=g\left(\mathbf{x}\left|\boldsymbol{\uptheta} \right.\right)+N\left(0,{\sigma}^2\right). $$

The objective of the modeling effort is typically to minimize the prediction error in regard to *y* and to recover the underlying parameters **θ** of the process as faithfully as possible.

The precise/not precise (PNP) model is based on the assumption that there is, for each response, a certain probability *λ* that an error will occur and, conversely, the probability (1 − *λ*) that an error will not occur. If *B* is a Bernoulli random variable with probability *λ*, each estimate *y* given some function *g*(**x**|**θ**) is defined by

2 $$ \left.y\right|\left(B=b\right)=\left\{\begin{array}{c}g\left(\mathbf{x}\left|\boldsymbol{\uptheta} \right.\right)+N\left(0,{\sigma}^2\right),b=1\\ {}g\left(\mathbf{x}\left|\boldsymbol{\uptheta} \right.\right),\kern4.75em b=0\end{array}\right.. $$

For Intuition(B) we have *λ* = 1 and ubiquitous Gaussian noise perturb the output of the model (in which case the model coincides with the standard model in Equation 1). In the case of Analysis(B), *λ* is presumably a small but nonzero number (no one is perfect, after all).

It is, however, impractical to define precise responses by a point estimate, because it does not supply a suitable probability density function,^1^ and thus preclude maximum likelihood estimation. Instead, it is prudent to introduce some narrow but integrable distribution as the definition of a precise response. Though there are many different distributions that could potentially fulfil this role, we have found that, for the purpose of the experiments included in this study, precise responses are best modeled by a very narrow Gaussian distribution, so that

3 $$ \left.y\right|\left(B=b\right)=\left\{\begin{array}{c}g\left(\mathbf{x}\left|\boldsymbol{\uptheta} \right.\right)+N\left(0,{\sigma}^2\right),b=1\\ {}g\left(\mathbf{x}\left|\boldsymbol{\uptheta} \right.\right)+N\left(0,{\tau}^2\right),\kern0.5em b=0\end{array}\right.. $$

This formulation uses the parameter *τ*, which specifies the width of the distribution defining a precise response. Therefore, *τ* should not be understood as an estimate of error, but rather a definition of the range within which the model will “accept” a precise response; a technical parameter rather than an empirical one. Because the aim of the PNP model is specifically to delineate (in theory) error-free responses, *τ* should obviously be bounded to very low values relative to *σ* , or alternatively set to a specific value that represents the appropriate precision for the analysis at hand. Note, however, that the most appropriate definition of *τ*, or its associated distribution, will be dependent on the structure of the task and the nature of the cognitive process one aims to capture. Because we are now dealing with two Gaussian functions (albeit one which is very narrow), the PNP model in practice constitute a mixture model of two Gaussian distributions, and it is possible to define a joint probability density function so that

4 $$ {\displaystyle \begin{array}{l}f\left(\left.y\right|\lambda, g,\mathbf{x},\boldsymbol{\uptheta}, \sigma, \tau \right)=\lambda f\left(\left.y\right|g,\mathbf{x},\boldsymbol{\uptheta}, \sigma \right)+\left(1-\lambda \right)f\left(\left.y\right|g,\mathbf{x},\boldsymbol{\uptheta}, \tau \right)\\ {}\\ {}=\frac{\lambda }{\sqrt{2{\pi \sigma}^2}}\exp \left(-\frac{{\left(y-g\left(\mathbf{x}|\boldsymbol{\uptheta} \right)\right)}^2}{2{\sigma}^2}\right)+\frac{\left(1-\lambda \right)}{\sqrt{2{\pi \tau}^2}}\exp \left(-\frac{{\left(y-g\left(\mathbf{x}|\boldsymbol{\uptheta} \right)\right)}^2}{2{\tau}^2}\right).\end{array}} $$

### Comments

The PNP model allows for the value of the function *g*(**x**|**θ**) to be sampled from either a precise or a nonprecise distribution, with sampling parameter *λ*. Crucially, the estimates of **θ** are conditional on this assumption. When data involves homogeneous Gaussian error the PNP model reduces to a conventional Gaussian likelihood function (because *λ* = 1). However, when some configuration of the parameters **θ** exists for which a significant proportion of precise responses is observed in relation to the function *g*(**x**|**θ**), the PNP model is more likely to find these parameter values and is less affected by biases contingent on the error distribution.

We acknowledge two potential caveats. First, when the response variable is discrete with few values there is a risk that participants make precise judgments “by chance,” in which case an estimate of *λ* may become biased and the error rate underestimated (see Appendix A for potential corrections). Second, if the function *g*(**x**|**θ**) is incorrectly specified, and the data derives from precise execution of a different process, this may lead to the estimate of *λ* becoming biased in the opposite direction and the error rate overestimated. Theory dependence is, of course, inherent to all cognitive modeling, and when we apply the PNP model to data, we mitigate this issue by also ascertaining that the model accounts for most of the systematic variance in the data.

## Illustrative examples and model recovery

Aside from prediction accuracy, two typical desiderata in cognitive modeling is that the procedure should correctly identify the cognitive process that best accounts for the data—for example, an Intuition(B) or an Analysis(B) process—and that it should identify the underlying parameters **θ** of the process as accurately as possible (e.g., the relative weighting of the cues). In the following section, we illustrate these requirements in two different ways. First, we provide two examples based on empirical data from Experiment 2 of the current article, which illustrate the application of the PNP model. Thereafter, we report a model recovery analysis that demonstrates the model’s ability to correctly recover the parameters of (simulated) data with a known generative model.

### Examples

These examples illustrate the application of the PNP model to data from two participants in Experiment 2 (reported below) and contrast this with conventional regression modeling. The two participants both performed a willingness-to-pay task, where they assessed the amount they were willing to pay (in Swedish Crowns: SEK) for each of 32 lotteries on the form .XX probability to win YY SEK, otherwise nothing (0 SEK). For each of the two participants, *g*(**x**|**θ**) consists of the expected value of the lottery in question, adjusted by the parameters **θ** = [α, β], so that *g*(**x**|**θ**) = α + β(probability × monetary reward). We report the maximum likelihood parameter estimates α, β, and σ, from the PNP model and from a standard regression model, as well as the maximum likelihood estimate of λ for the PNP model. For both participants, τ = 1 ×10^-3^.

**Fig. 1:**
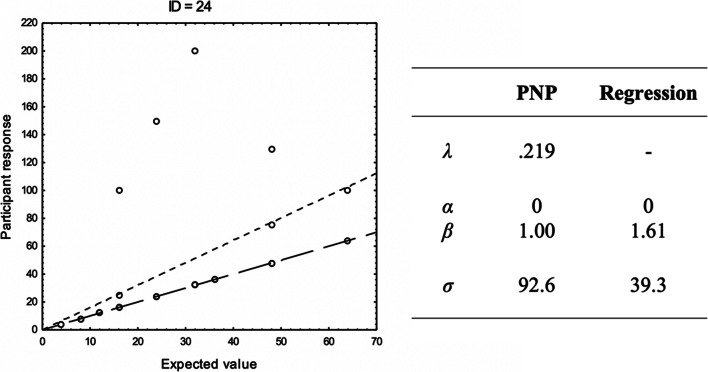
The responses by participant ID = 24 in a willingness-to-pay task in Experiment 2 reported below plotted against the expected value, with estimated parameter values from the PNP model and a conventional regression model. A line representing the predictions of the regression model (thinner line) and a reference line *x* = *y* (thicker line) is included in the graph. The predictions by the PNP model coincide with the reference line. Note that some data points overlap

Participant ID = 24 (see Fig. 1) apparently calculated expected values of the proposed lotteries, resulting in a majority of values (25 / 32) perfectly in sync with the expected values of the lotteries and a certain amount of large errors (7 / 32). Consequently, the PNP model indicates that most responses are perfect executions of the expected values (i.e., that α = 0, β = 1), but that there is a certain probability (λ = .219) that errors with high standard deviation (σ = 92.6) occur. Note that the λ parameter represents the proportions of nonprecise responses relative to the model, because 7 / 32 = .219. As suggested by the low λ, the error distribution around the predictions by the PNP model is leptokurtic and the null model of a homogenous Gaussian error distribution can safely be rejected (Kolmogorov–Smirnov K-S test, *p* < .001). 2

The process described by standard regression modeling indicate that all responses are affected by errors with a lower (though still substantial) standard deviation (σ = 39.3) and that the expected value is over-estimated (i.e., multiplied by a factor of approximately 1.61). As suggested by a best fitting value of λ = .219, the PNP model provides better fit to data than the standard regression model, identifying (correctly, we believe) the underlying process as an explicit calculation of the expected value, marred by occasional and large errors.

**Fig. 2:**
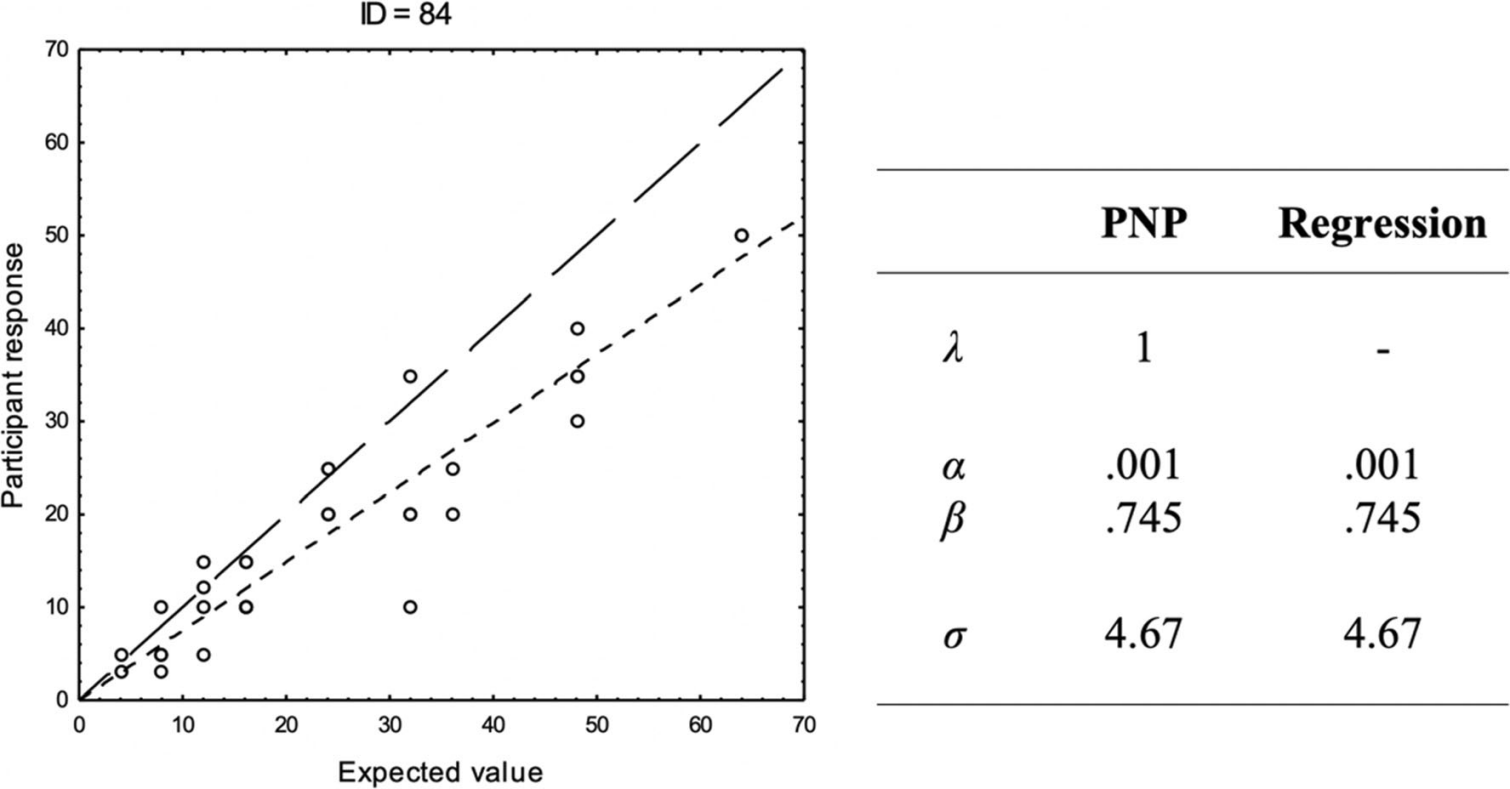
The responses by participant ID = 84 in a willingness-to-pay task in Experiment 2 reported below plotted against the expected value, with estimated parameter values from the PNP model and a regression model. A line representing the predictions of both models (thinner line) and a reference line *x* = *y* (thicker line) is included in the graph. Note that some data points overlap

Participant ID = 84 (see Fig. 2) applies an Intuitive(B) strategy, preferring to pay less than the lotteries’ expected value but apparently not using an exact calculation. The parameters α and β represent a negatively biased expected value, errors are small, and because no response correspond exactly to the estimated function the value of λ is equal to 1. The PNP model here reduces to the same equation as the standard regression model, and the parameter estimates for the two models are the same. The errors around the predictions by the PNP model appear normal and there is no evidence that the null model of a homogenous Gaussian error should be rejected (K-S test, *p* = .463). When adjusted for free parameters, the standard regression model yields marginally better fit than the PNP model, but both models provide the same view of the process: The judgment is an underestimation of the expected value perturbed by homogenous error.

### Model recovery

**Fig. 3:**
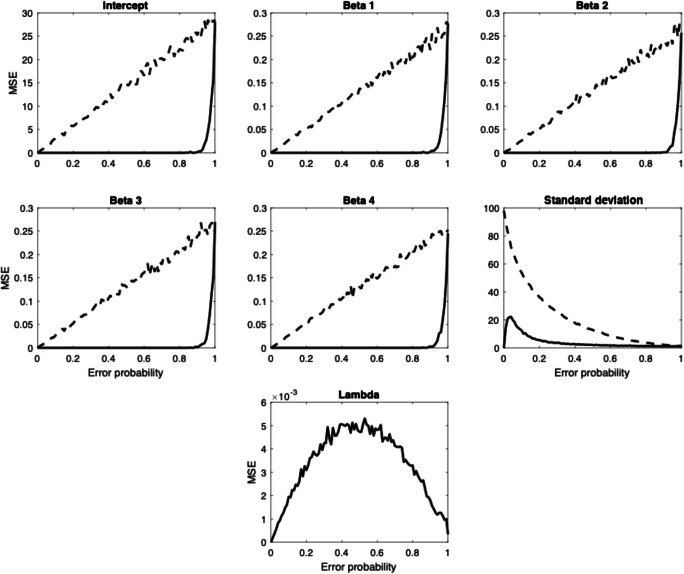
Mean squared error for all parameters for the standard regression model (dashed line) and the PNP model (solid line) for each error probability

The PNP model was also applied to simulated data intended to be representative of previous applications of cognitive modeling to multiple cue judgments (see Karlsson, Juslin, & Olsson, 2007; for similar studies, see, e.g., Hoffmann, von Helversen, & Rieskamp, 2014, 2016; Juslin, Karlsson, & Olsson, 2008; Juslin, Olsson, & Olsson, 2003; Little & McDaniel, 2015; Pachur & Olsson, 2012; Platzer & Bröder, 2013; von Helversen, Mata, & Olsson, 2010; von Helversen & Rieskamp, 2009). In this task, participants learn to predict a continuous criterion *y* from four continuous cues *x*_1_…*x*_4_ based on outcome feedback training. It is assumed that the participants have abstracted the linear weights of each cue, which are integrated into a judgment $\hat{y}$ of the criterion.^3^ Data were simulated by sampling cue values *x*_1-4_ from a uniform distribution [0, 10] and computing a response $\hat{y}$ from the cue values according to a linear model,

5 $$ \hat{y}=\alpha +{\beta}_1{x}_1+{\beta}_2{x}_2+{\beta}_3{x}_3+{\beta}_4{x}_4, $$

with α = 50, β_1_ = 4, β_2_ = 3, β_3_ = 2, and β_4_ = 1. One hundred and one simulations were performed, with probabilities of *λ* = (0, .01, .02, … 1) that responses were perturbed by noise generated from a normal distribution with mean μ = 0 and standard deviation σ = 10. For each of the simulations, data from *N* = 1,000 simulated participants each with a sample size of *n* = 50 were generated, corresponding to a (fictive) situation where the models are applied to data from 1,000 participants each having made 50 multiple cue judgments. In each simulation, both a conventional regression model equivalent to Equation 1 (Reg.) and the PNP model were applied to the data, both of which had access to the correct model structure (Equation 5), but not to the correct parameter values. The PNP model should, in addition to estimating the parameter values in Equation 5, correctly estimate *λ*—that is, correctly recover the proportion of responses that were perturbed by noise. Figure 3 plots the mean square error (MSE) of the estimated versus the generating parameters for the two models for each error probability, with *λ* only being estimated by the PNP model. The estimated value of, and overall means for, each of the parameters α, β_1-4_, and *σ* are presented in Table 1.

**Table 1:** Grand means of parameter estimates after applying respectively a conventional regression model and the PNP model to simulated data

	*α* (50)	*β*_1_ (4)	*β*_2_ (3)	*β*_3_ (2)	*β*_4_ (1)	*σ* (10)
PNP	50.0	4.00	3.00	2.00	1.00	9.78
Reg.	50.0	4.00	3.00	2.00	1.00	6.19

*Note.* True parameter values are presented in parentheses below the names of the parameters

The MSE plotted in Fig. 3 demonstrates that the PNP model recovers the α and β_1-4_ parameters with much higher precision than the regression model, unless error probability is either very low or very high, in which case the models have the same precision. The apparently large discrepancy in MSE for error probabilities in between these values follows from the fact that, when there are a significant number of precise values for the PNP model to find, these values are generally consistent with the parameter values of the generating model, and thus the exact parameters will be extracted. For the regression model, by contrast, any error in data will always add error to the parameter estimates. Importantly, even though both models correctly identify the *σ* parameter when the error probability is *p* = 1, only the PNP model will successfully recover the *σ* parameter when the error probability is *p* < 1, while the Reg. model’s estimate of *σ* will be biased. Additionally, the PNP model will recover the *λ* parameter with very high precision (MSE < .006). In sum: When the underlying generative model is known, as in model recovery, the PNP model very accurately identifies if the process is an Intuition(B) or an Analysis(B) process. In the latter case, it will also give much more accurate parameter estimates than a regular regression model that assumes a homogenous Gaussian noise distribution.

## Experiment 1: Revisiting conceptual and perceptual tasks

**Fig. 4:**
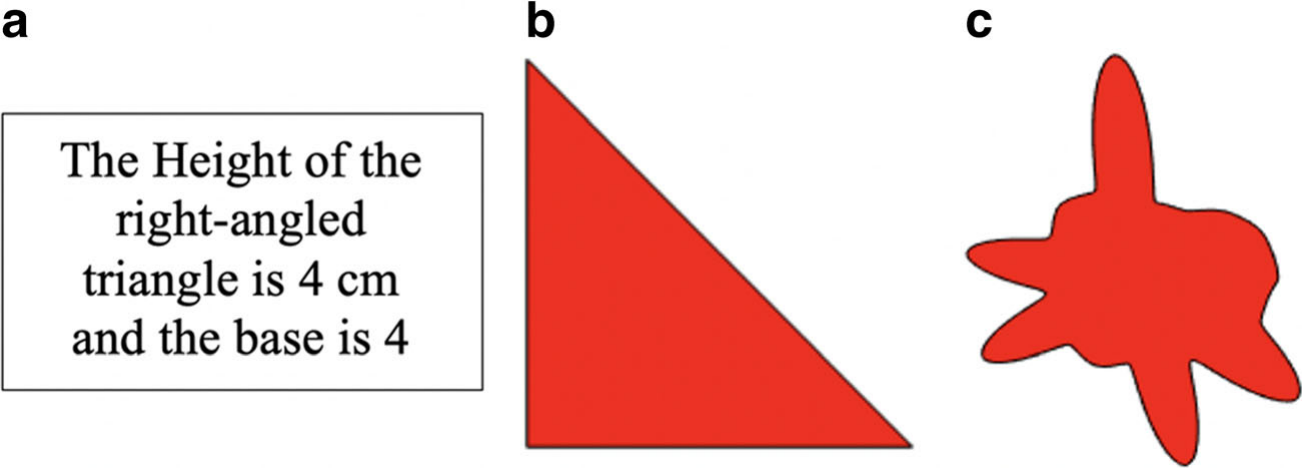
Examples of stimuli in the condition with a conceptual triangle (**a**), the condition with a visual triangle (**b**), and the condition with the blob shape (**c**). In all conditions, the participants were asked to estimate the area covered by the object in square centimeters (cm^2^)

The purpose of Experiment 1 was to replicate Brunswik’s claims about error distributions in the original context of perceptual and conceptual processes and to demonstrate that the PNP model faithfully identifies and captures Brunswik’s distinction. The participants made area judgments in three conditions: (i) A conceptual task, where they used numeric measures of the base and the height of a triangle to infer its area. (ii) A perceptual task, where the participants encode the base and the height of right-angled triangles with unaided visual perception and infer the area. (iii) A perceptual task, where the participants inferred the area covered by irregular shapes (or “blobs”) with the same area as the triangles in the two first conditions (see Fig. 4 for illustrations of the stimuli).

We expected the majority of our participants (undergraduate students) to know the equation relevant for calculating the area of a triangle and to make use of this equation in the conceptual condition in a manner consistent with Analysis(B) processes. Conversely, the irregular blobs lacked obvious characteristics on which to base explicit numerical calculations, so we expected these participants to engage in Intuition(B) processes. In the visual triangle condition, the visual encoding of the sides of the triangle were likely to be perturbed by a sensory noise, but the participants could potentially apply the numerical equation on their noisy estimates. This would leave the situation open for blends of Analysis(B) and Intuition(B) processes.

Thus, we expected to replicate Brunswik’s (1956) claim that Intuition(B) processes in the perceptual tasks produce a Gaussian error distribution, and that the Analysis(B) processes in the conceptual task produce a leptokurtic error distribution. Moreover, the PNP model should accurately identify the two processes in a corresponding manner, by indicating a *λ* close to 1 for Intuition(B) processes and a low, but usually nonzero, *λ* for Analysis(B) processes. For Analysis(B), the process parameters α and β should also identify that most responses correspond with numerically exact rather than approximate calculations of the area of the triangle.

### Method

#### Participants

Forty-six participants (27 females, 18 males, and 1 nonbinary individual) ranging in age from 19 to 74 years (*M* = 29.13, *SD* = 9.60) were recruited through public advertisement at various places at Uppsala University. Compensation was awarded in the form of a cinema voucher or (for students at the Department of Psychology) course credit.

#### Design

The experiment had a between-subjects design, with *task content* (numerically presented triangle, visually presented triangle, and visually presented blob) as independent between-subjects variables. The dependent measure was the participants’ judgments of the area (in cm^2^) of the presented stimulus.

#### Material

The base of the triangle could take five values (4, 5, 6, 7, 8), and the height could take five values (4, 5, 6, 7, 8). A complete 5 × 5 factorial combination produced a total of 25 items. All visually presented triangles were right-angled triangles, with the right angle in the bottom-left corner. The numerically presented triangle was described as a right-angled triangle. Blobs were created in MATLAB by sampling irregular shapes with an area deviating at most 0.1 cm^2^ from each triangle counterpart (see Fig. 4 for an illustration of the stimuli, and Appendix B for a detailed account of the instructions to the participants).

#### Procedure

The participants conducted the experiment in separate computer booths at the Department of Psychology at Uppsala University under supervision of an experiment-leader. Participants were randomly assigned to each of the between-subjects conditions. Each of the 25 items were presented in a random order, then the same 25 trials were administered a second time, again in random order, resulting in a total of 50 trials per participant. Trials were presented one at a time, and the participants recorded their estimate for each trial before moving on to the next. No response feedback was provided.

#### Application of the PNP model

The PNP model was fitted to individual participant data with a function *g*(**x**|**θ**) that capture both biased and unbiased (i.e., α = 0, β = 1) area estimates,

6 $$ Estimate=g\left( Area|\alpha, \beta \right)=\alpha +\beta \times Area. $$

The *τ* parameter was set to represent a standard deviation three decimals below the variation of the response variable (i.e. *τ* = 1 × 10^-4^). This means that the value of *τ* lies well below the precision with which participants are likely to report their responses, especially given that the instructions asked for estimates with up to one decimal, and only precise responses are likely to be within the tolerance.^4^

#### Model fit

Parameters were estimated by maximum likelihood estimation. In the context of model selection, Bayesian information criterion (BIC) was used to identify the best model fit (see Raftery, 1995). Because BIC contributes no information on the absolute fit of a model, we also report the standard measure of “explained variance,” adjusted *R*^2^. Furthermore, if we identify the correct model, all the systematic variance in data should be accounted for, and all the residual noise should be random. We therefore also report a Saturation Index (SI), which we define as

7 $$ SI={R}^2/\rho, $$

where *ρ* is the reliability coefficient, or proportion of true variance in data, as estimated by the test–retest reliability when participants perform each judgment twice. SI will approach 1 if the model accounts for all the true variance in data, while a low SI suggests that there is nontrivial residual systematicity in the data that the model fails to explain. Applying the PNP model thus entails not only finding the best fit as measured by BIC but also a sufficiently high SI.

To exemplify, if there is 90% systematic variance and the model accounts for 90% of the total variance, the model “saturates” the data in the sense that it accounts for all the systematic variance and SI = 1. If a model accounts for only 50% of the variance in data, but there is 90% systematic variance, then SI = .56, suggesting that it fails to capture the correct function.

### Results and discussion

Descriptive statistics for Experiment 1 in terms of participant’s area estimates in the experimental task, correlation coefficients between response and criterion, RMSD from an error-free calculation of triangle area, and median response times can be found in Appendix C.

#### Distribution of errors

**Fig. 5:**
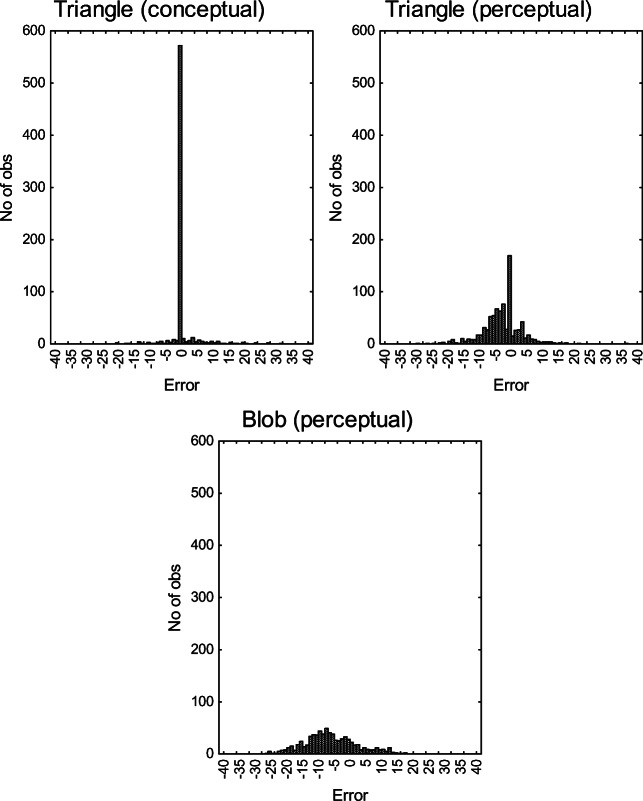
Histograms of error distributions defined as deviations from the objective area

Figure 5 illustrates the error distributions of responses in relation to the criterion in the three conditions of Experiment 1. As originally claimed by Brunswik, it is clear that the error distribution for the conceptual task with numerical triangles is strikingly leptokurtic, while the error distributions in the two perceptual tasks have a Gaussian shape, with a slightly more pronounced “peak” in the perceptual triangle condition.

#### Individual-level modeling

**Fig. 6:**
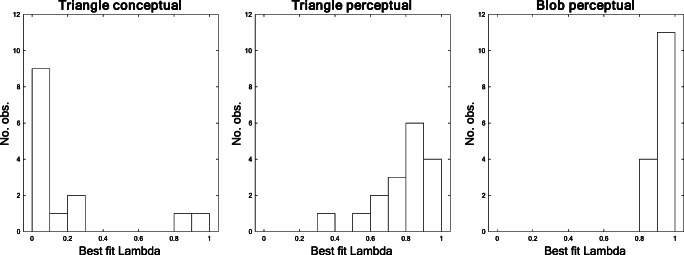
Histograms of estimated *λ* parameter values for each participant in each condition

The distribution of the best fitting *λ* parameter for each individual participant indicates that participants typically had either low (*λ* < .1) or high (*λ* > .8) values of *λ* (see Fig. 6). We take these distributions to suggest that, in the specific tasks addressed in this experiment, the processes used by the participants tend to spontaneously cluster into two fairly distinct categories, consistent with Intuition(B) and Analysis(B).

Table 2 reports median parameter estimates (λ, α, β, & σ) and median fit indices (Adj. *R*^2^, & SI) for individuals in each condition. The SI in Table 2 indicate that the model described in Equation 6 accounts for almost all the true variance in data for the conceptual and perceptual triangles, and most of the variance in data for the area estimates of blobs. Due to the skewed distributions, a Kruskal–Wallis ANOVA was used to confirm a statistically significant difference in the estimates of the *λ* parameter between conditions, *χ*^2^(2) = 26.5 *p* < .001, indicating that participants in the conceptual condition are better described by an Analysis(B) process while participants in the perceptual conditions are better described by an Intuition(B) process. Indeed, the median parameters in Table 2 confirm that the most frequent responses for the participants with low *λ* were a perfectly error-free calculation of the area (α = 0, β = 1). As suggested by the distributions in Fig. 5 and the median estimated *λ* in Table 2, the null model of homogenous Gaussian residuals was rejected beyond an α level of .05 (K-S tests) for 93% (13/14) of the participants in the conceptual triangle condition; for 47% (8/17) of the participants in the perceptual triangle condition; but only for 20% (3/15) of the participants in the perceptual blob condition.

**Table 2:** Compilation of median parameter estimates for each condition in Experiment 1

Condition	Parameter estimates				Model fit	
	λ	*α*	β	σ^*^	*R*^2^	SI
Area of triangle (conceptual) *n* = 14	0.05	0	1	6.36	0.954	0.994
Area of triangle (perceptual) *n* = 17	0.86	0	1	4.15	0.728	0.942
Area of blob (perceptual) *n* = 15	0.92	2	0.5	2.67	0.578	0.875

*Note that *σ* is calculated only on errors that actually occurred, and thus individuals with zero error variance is excluded from the median estimation, SI = saturation index

#### Magnitude of errors

The error magnitudes, as measured by the standard deviation *σ*, were correlated with the estimates of the *λ* parameter to evaluate if errors in Analysis(B) processes are larger in magnitude than errors in Intuition(B) processes. Because we are concerned with the relative magnitude of error, participants who had no error variance (three individuals) were excluded. A Bayesian Kendall’s tau indicated strong evidence that the error magnitude is negatively correlated with lambda, *r*_*τ*_ **= −.**336 BF_10_ = 26.4, thus confirming Brunswik’s claim that Analysis(B) are associated with larger errors than Intuition(B).

#### Conclusions

The results of Experiment 1 confirm Brunswik’s claims about the error distributions in perceptual versus conceptual tasks. Importantly, the results verify that the PNP model can successfully use them to identify the Analysis(B) processes in the conceptual task and recover the expected parameters for correctly calculating the exact area of the triangle (i.e., α = 0, β = 1), as well as capture the increasingly important components of Intuition(B) processes when the input is nonsymbolic and there is no applicable rule. In these tasks, the results also confirm Brunswik’s assertion that Analysis(B) processes often give rise to larger errors.

The median estimate of *λ* in the perceptual triangle condition was .86, while the median parameter estimates of α and β were consistent with correctly calculating the exact area of the triangle. Although many participants in this condition estimate the exact area of the triangle, most responses deviate from this prediction. Apparently, participants in the perceptual triangle condition tried to calculate the exact area of the triangle by using the analytical rule for area calculation (which they should be familiar with), but due to neural noise in the visual process, most of their area calculations are nonprecise. Presumably, many participants rounded their length estimations to integers. Because the stimulus lengths were indeed integers, this would result in occasional estimates being consistent with the exact lengths, and thereby increasing the rate of error-free area estimations, despite neural noise in the visual process. This conclusion is validated by both the error distributions presented in Fig. 5, showing a slight “spike” in the perceptual triangle condition, as well as the testing of the residuals from the model predictions providing evidence against the null hypothesis of homogenous Gaussian error for 47% of the participants in the condition.

## Experiment 2: Application to a range of conceptual tasks

In Brunswik’s (1956) original experiment, he compared a strictly perceptual task to a strictly conceptual one, but as noted by Hammond (1988), Intuition(B) need not be associated only with perceptual tasks: “Numbers can produce hasty, intuitive judgments based on a minimum of analysis, and pictures can become the subject of endless analysis” (p. 4). The purpose of Experiment 2 was to apply the PNP model to a wider range of tasks, validating that both Analysis(B) and Intuition(B) are also present in conceptual tasks that do not emphasize sensory encoding and involve symbolic (numerical) cues. In Experiment 2, participants therefore made judgments in one of six different domains, for which there existed an identical multiplicative algorithm by which the judgments could be made. The domains ranged from those where a fairly self-evident analytic algorithm exists (e.g., in an abstract math problem or a version of the area task used in Experiment 1) to those where the multiplicative integration is less obvious (e.g., the performance-assessments and the willingness to pay for lotteries).

In contrast to Experiment 1, we compared two alternative algorithms. We assumed that the participants either relied on the multiplicative reference function suggested by the content, or that they fell back on the default of linear additive integration often observed in cue integration tasks (Brehmer, 1994; Karelaia & Hogarth, 2008; Juslin, Nilsson, & Winman, 2009). The purpose was therefore to use the PNP model not only to ascertain if the cognitive process could be identified as Analysis(B) or Intuition(B), but also to identify the integration rule used.

Our hypothesis was that multiplicative cue integration should primarily draw on Analysis(B) processes, because the multiplicative integration should primarily occur when the participants can draw on mental calculation according to declaratively known rules (as predicted by research on numerical cognition; e.g., Dehaene, 2011). In the absence of guidance of such declaratively known rules (in the performance and potentially WTP conditions), we expected the participants to either default to additive integration by Intuition(B) processes, as often observed in multiple-cue judgments (e.g., Brehmer, 1994; see also Experiment 3 below) or to uphold multiplicative integration, but in an approximate manner consistent with Intuition(B), given the absence of an exact integration rule (as suggested by Anderson, 1996, 2008). Thus, to the extent that participants solved the task by explicit numeric calculation of the multiplicative reference function, the PNP model should identify this as an Analysis(B) process with process parameters identifying that the typical response is indeed an error-free computation of the reference function (α = 0, β = 1). In addition, we aimed to explore if judgment errors in the Analysis(B) process are larger in magnitude, as observed by Brunswik and replicated in Experiment 1.

### Method

#### Participants

Ninety participants (55 females, 34 males, and 1 nonbinary individual) ranging in age from 19 to 53 years (*M* = 23.93, *SD* = 4.86) were recruited through public advertisement at various places at Uppsala University. Compensation was awarded in the form of a cinema voucher or (for students at the Department of Psychology) course credit.

#### Design

The experiment was an 6 × 3 mixed factorial design, with *task content* as an independent between-subjects variable (abstract mathematics, area, performance, speed, expected value, and willingness to pay) and *response variable*^5^ as a within-subjects variable (product, multiplicand 1 [M_1_], and multiplicand 2 [M_2_]). These contents either have an obvious strong normative rationale for multiplicative integration (i.e., abstract mathematics, area, speed, and expected value) or have been empirically shown to involve multiplicative integration in previous research (i.e., motivation and willingness to pay; for a review, see Anderson, 1996, 2008). We therefore compare the judgments in all six domains to a common multiplicative reference function, although obviously the normative rationale for multiplicative integration is considerably stronger with some of the contents than with others. The dependent measure was the participants’ judgments of the criterion.

#### Material

M_1_ consisted of four values (.2, .4, .6, & .8), and M_2_ consisted of four other values (20, 40, 60, & 80). A complete 4 × 4 factorial combination produced a total of 16 items. The multiplicative reference functions that we assumed for inferring M_1_, M_2_, or the product were:

8 $$ {\displaystyle \begin{array}{l}{M}_1=\frac{Product}{M_2}\\ {}{M}_2=\frac{Product}{M_1}\\ {} Product={M}_1\times {M}_2\end{array}}. $$

Below, we provide task instruction for the area task, the performance task, and the speed task for each direction of inference (M_1_, M_2_, Product; see Appendix D for the instructions for the Willingness to Pay, Expected Value, and Mathematics).

- *Area of a plank.* Your task is to estimate the following lengths and areas correctly as fast as possible. The numbers involved may involve either proportions (e.g., .50) or integers (e.g., 50).

The width of a plank is .20 meters and its length is 20 meters. What area in m^2^ is covered by the plank? ______

The area covered by a plank is 4 m^2^. The length of the plank is 20 meters. What is the width of the plank? ______

The area covered by a plank is 4 m^2^. The width of the plank is .20 meters. What is the length of the plank? ______

- *Motivation, ability, and grade*. Your task is to estimate the likely motivation, ability, and grade on a test for the following students as fast as possible. The motivation of a student is measured as a proportion of the student’s full motivation (e.g., .50 is 50% of full—100%—motivation). The ability is measured by a score between 0 and 100, and the grade received by each student on the test is a number between 0 and 64.

The motivation of a student is .20, and the ability score of the student is 20: What is your best guess for the likely grade of this student? ______

The grade obtained by the student in the test was 4. The ability score of the student is 20. What is your best guess for the likely motivation of this student? ______

The grade obtained by the student in the test was 4. The motivation of the student is .20. What is your best guess for the ability score of this student? ______

- *Speed, distance, and time*. Your task is to estimate the following speeds, distances, and times as correctly and as fast as possible.

Something moves at a speed of .20 meters per hour. What distance in meters will have been covered after 20 hours? ______

Something moves a distance of 4 meters. This movement takes 20 hours. What is the speed of the movement in terms of meters per hour? ______

The speed of the movement of something is .20 meters per hour. How long will it take it to go a distance of 4 meters? ______

#### Procedure

The experiment was performed at Uppsala University, and participants conducted the experiment in separate computer booths. The online survey software Lime-Survey was used to collect the data. Participants were randomly assigned to each of the between-subjects conditions. Each of the 16 items were administered once for each response variable in random order, for a total of 48 trials, then those 48 trials were administered a second time, again in random order, resulting in a total of 96 trials per participant. Trials were presented one at a time, and the participants recorded their estimate for each trial before moving on to the next. No response feedback was provided.

#### Application of the PNP model

The PNP model was fitted to individual participant data with two functions *g*(**x**|**θ**) that capture multiplicative and additive cue integration rules, respectively. The multiplicative function was the linearly adjusted product (or dividend). When M_1_, M_2_ or the product were estimated the multiplicative function was defined respectively as

9 $$ {\displaystyle \begin{array}{l}{M}_1=g\left( Product,{M}_2\left|\alpha, \beta \right.\right)=\alpha +\beta \left(\left(\frac{Product}{M_2}\right)\right),\\ {}{M}_2=g\left( Product,{M}_1\left|\alpha, \beta \right.\right)=\alpha +\beta \left(\left(\frac{Product}{M_1}\right)\right)\\ {} Product=g\left({M}_1,{M}_2\left|\alpha, \beta \right.\right)=\alpha +\beta \left({M}_1\times {M}_2\right),\end{array}} $$

which allowed us to model both biased and unbiased use of multiplication and division.

The additive function consisted of a linear combination of the data values, defined as

10 $$ y=g\left({x}_1,{x}_2\left|\alpha, {\beta}_1,{\beta}_2\right.\right)=\alpha +{\beta}_1{x}_1+{\beta}_2{x}_2, $$

in which *y* denotes one of the three response variables (M_1_, M_2,_ or the product) and *x*_1_ and *x*_2_ denotes the two other variables. Model parameters were estimated separately for each response variable.

Because the variation in criterion values differed across response variable (see Material), the value of *τ* for each response variable was set to represent a standard deviation three decimals below this variation (i.e., *τ* = 1 ×10^-4^ for M_1_, *τ* = 1 ×10^-2^ for M_2_, and *τ* = 1 ×10^-3^ for the product). In other respects, the models were fitted in the same manner as in Experiment 1.

### Results and discussion

Ehe descriptive statistics for the judgments made with each of the six contents in terms of the median judgments, the median correlation with the multiplicative reference function, the median root mean square deviation (RMSD) from the multiplicative reference function, and the median response times are summarized in Appendix E. Generally, the judgments were well described by the multiplicative reference function for the contents Math, Area, Speed, and Expected Value (EV), but less so for Performance and Willingness to Pay (WTP).

#### Individual-level modeling

**Fig. 7:**
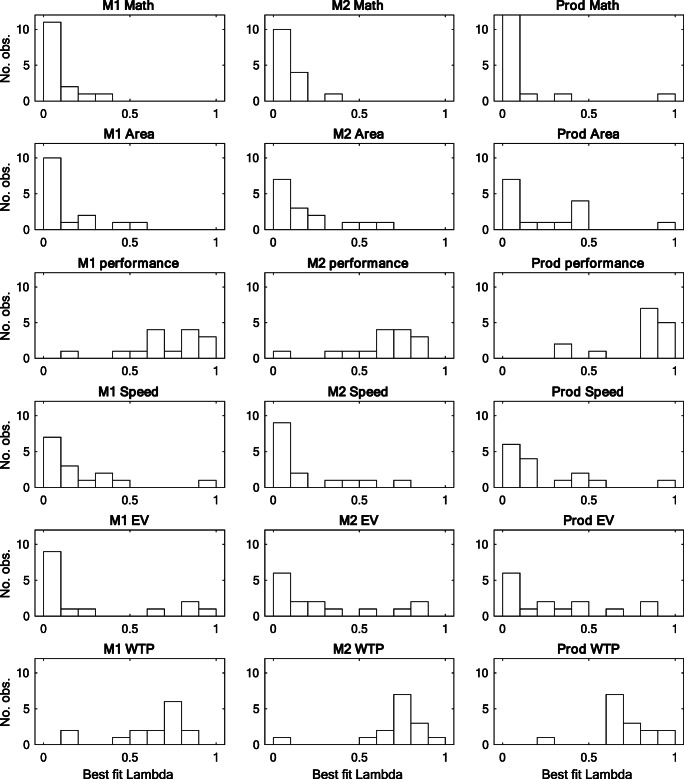
Histograms of estimated *λ* parameter values for the dominant PNP-process model for each participant and for each condition

The PNP model was applied to the individual participant data using both the multiplicative and additive functions, and each participant was categorized as either multiplicative or additive based on which algorithm received the lowest BIC. The *λ* estimates for the best fitting models are presented in Fig. 7. To reiterate, if participants approach a task with Analysis(B) type processes we expect errors to be few (i.e., low *λ*) and therefore the error distribution to be leptokurtic, while Intuition(B) type processes are associated with ubiquitous errors (i.e., *λ* = 1) and an error distribution that is Gaussian in shape. The overall bimodal pattern observed in Experiment 1 was observed also across the six tasks used in Experiment 2, but, as with the perceptual triangle condition in Experiment 1, the right-most peaks of the distributions are somewhat less close to the edge of the scale as one might expect if participants were relying strictly on Intuition(B) processes. To some extent, this appears to be a side effect of the small number (4) of even values used as criterion for M_1_ and M_2_, in combination with the within-subjects design. By rounding the responses to even values, the participants will sometimes produce exactly correct responses, and this tendency is reinforced by the within-subjects design, where they may have experienced these four values in a previous condition (e.g., if they assess the product before they assess the M_1_ and M_2_).

Table 3 report median fit indices (Adj. *R*^2^, & SI) for the best fitting models, which support three conclusions. First, the PNP models generally explain more variance when applied to the Math, Area, and Speed contents (most *R*^2^ > .9), whereas the variance explained is lower for Performance, EV, and WTP (all *R*^2^ < .9). Second, the lower *R*^2^ for EV does not derive from a failure to capture the true variance in the data (SI > .9), but from more noise in data (e.g., when assessing the product for EV, the median *R*^2^ is only .553, but the median SI is .970, suggesting that almost all the true variance is captured by the model). Third, there is a direction sensitivity for Performance. SI is high in all directions for all conditions except for Performance, where SI is lower when participants infer M_1_ than when they infer M_2_ and the product. Thus, although the SI indicates that our algorithms capture the process well in most of the conditions, it also clearly signals that we fail to capture the process behind Performance judgments regarding M_1_.^6^

**Table 3:** Median adjusted *R*^2^ and SI (saturation index) for the best fitting model (determined by BIC) for each of the conditions in Experiment 2

Content	M_1_		M_2_		Product	
	*R*^2^	SI	*R*^2^	SI	*R*^2^	SI
Math	.955	.995	.929	.986	.998	1.00
Area	.937	.994	.722	.952	.922	.988
Performance	.021	.508	.655	.883	.798	.902
Speed	.861	.989	.919	.982	.960	.988
EV	.615	.994	.736	.999	.553	.970
WTP	.627	.907	.644	.832	.740	.873

Median *λ* estimates, the number of individuals best fitted by the multiplicative versus the additive model, and median parameter values for each best fitting model in each condition are summarized in Table 4. We clearly see that, for the Math, Area, Speed, and EV conditions, the dominant integration rule was consistent with Equation 8 (multiplication), and with a low proportion of error (median *λ* ≤ .25). The Performance and WTP conditions were more varied, in terms of both parameter estimates and best fitting models, with clear evidence for multiplication only when the participants inferred M1 in the WTP condition. Otherwise, Bayesian binomial test provided nu support for a dominating additive or multiplicative integration rule for Performance (M_1_: BF_10_ = 1.5, M_2_: BF_10_ = .318, Prod: BF_10_ = .318) or WTP (M_2_: BF_10_ = .409, Prod: BF_10_ = 1.5). Three Kruskal–Wallis ANOVAs confirmed that the differences in *λ* are statistically significant for each response variable—M_1_: χ^2^(5) = 42.3, *p* < .001, M_2_: χ^2^(5) = 42.1, *p* < .001, Prod: χ^2^(5) = 44.1, *p* < .001, and this difference was also confirmed when testing over participants best fitted by the multiplicative model only—M_1_: χ^2^(5) = 37.1, *p* < .001, M_2_: χ^2^(5) = 25.3, *p* < .001, Prod: χ^2^(5) = 39.7, *p* < .001. This confirms that both Analysis(B) and Intuition(B) type processes are present, and that the Intuition(B) processes are not only observed for the additive model.

**Table 4:** Compilation of overall median λ and the distribution of best fitting model for each response variable (within participant) in each condition (between participants), and median parameter estimates for best fitting multiplicative or additive model

		Median *λ*	Mult		Add	
	M_1_	.031	15	α = .000, β = 1.00, σ = .200, λ = .031	–	
Math	M_2_	.063	15	α = .000, β = 1.00, σ = 35.6, λ = .063	–	
	Prod.	.063	14	α = .000, β = 1.00, σ = 58.1, λ = .047	1	α = 100, β_1_ = 3.57, β_2_ = −.035, σ = 94.3, λ = .978
	M_1_	.063	15	α = .000, β = 1.00, σ = .279, λ =.063	–	
Area	M_2_	.156	15	α = .000, β = 1.00, σ = 22.8, λ = .156	–	
	Prod.	.125	14	α = .000, β = 1.00, σ = 15.7, λ = .109	1	α = 10.0, β_1_ = 3.47, β_2_ = −.035, σ = 9.46, λ = .953
	M_1_	.719	11	α = .000, β = 1.00, σ = 8.67, λ =. 688	4	α = 2.99, β_1_ = .196, β_2_ = .049, σ = 19.2, λ = .957
Performance	M_2_	.688	7	α = .000, β = 1.00, σ = 24.6, λ = .688	8	α = 8.33, β_1_ = 1.46, β_2_ = −20.0, σ = 15.2, λ = .688
	Prod.	.852	8	α = 17.0, β = .625, σ = 7.30, λ = .848	7	α = −.500, β_1_ = 32.5, β_2_ = .325, σ = 3.51, λ = .866
	M_1_	.156	15	α = .000, β = 1.00, σ = .210, λ = .156	–	
Speed	M_2_	.032	15	α = .000, β = 1.00, σ = 25.1, λ = .032	–	
	Prod.	.156	15	α = .000, β = 1.00, σ = 10.0, λ = .156	–	
	M_1_	.063	15	α = .000, β = 1.00, σ = .242, λ = .063	–	
EV	M_2_	.188	13	α = .000, β = 1.00, σ = 23.8, λ = .156	2	α = 5.99, β_1_ = 1.53, β_2_ = −11.1, σ = 111, λ = .842
	Prod.	.250	15	α = .000, β = 1.00, σ = 27.1, λ = .250	–	
	M_1_	.714	15	α = .233, β =.833, σ = .180, λ =. 714	–	
WTP	M_2_	.751	9	α = .000, β = 1.00, σ = 32.4, λ = .719	6	α = 22.5, β_1_ = 2.50, β_2_ = −.001, σ = 35.4, λ = .818
	Prod.	.692	11	α = 5, β = .625, σ = 4.70, λ = .688	4	α = −7.88, β_1_ = 25.0, β_2_ = .425, σ = 5.35, λ = .738

In alignment with the median estimated *λ* in Table 4, the null model of a homogenous Gaussian residual distribution was rejected beyond the conventional α level of .05 for most of the participants in the Math, Area, Speed, and Expected Value conditions (between 87% and 100% in all directions; K-S test). This null model was only rejected for about half or the participants in the Performance and Willingness to Pay conditions (between 13% and 60%, depending on the direction of inference). Importantly, in 73% of all the applications of the PNP model, the null model of a homogenous Gaussian residual distribution was rejected beyond the conventional α level of .05, suggesting that a homogenous Gaussian error is an incorrect specification of the error, which in turn may lead to biased parameter estimates (see the analyses for ID-24 in Fig. 1, above).

#### Error magnitude

As in Experiment 1, the error magnitudes were correlated with the estimates of the *λ* parameter. Participants with no error variance at all were excluded from the analysis. Contrary to in Experiment 1, Bayesian Kendall’s tau indicated no support for any negative correlations (M_1_: *r*_*τ*_ = .130, BF_10_ = .608; M_2_: *r*_*τ*_ **= −**.071, BF_10_ = .223; Prod: *r*_*τ*_ **= −**.151, BF_10_ = 1.03).

#### Conclusions

The results of Experiment 2 verify that the PNP model identifies parameters consistent with Analysis(B) in the Math, Area, Speed, and EV conditions, as would be expected if people applied the arithmetic of Equation 8. In these conditions the PNP model recovers exactly the multiplicative reference function invited by the contents (i.e., α = 0, β = 1). The Performance and WTP conditions are less straightforward to interpret, with median *λ* ≈ .75. We observed a similar result in the perceptual triangle condition in Experiment 1, apparently due to contributions of both Analysis(B) and Intuition(B) processes. This is potentially the case here as well but, as previously discussed (see Individual-Level Modeling section), it is equally likely to be a consequence of the experimental design as of the nature of the task. Indeed, the median parameter estimates for participants best fit by a multiplicative model for inferring M_1_ and M_2_ (Performance) and M_2_ (WTP) suggest that although most response were not precise, precise response were in line with the objective criterion.

Nonetheless, we do confirm that Intuition(B) processes are *present* and arguably *dominant* in the Performance and WTP conditions. Note that, for a given cue-integration rule (i.e., additive or multiplicative), the only difference between the Analysis(B) and Intuition(B) instantiations of the model refers to the assumptions about the shape of the error distributions. Yet the modeling results clearly separate most of the tasks addressed in Experiment 2 into tasks dominated either by Analysis(B) or Intuition(B) based on these differential assumptions alone. Importantly, the results also illustrate that the procedures we used in the PNP modeling also signal when the considered algorithms do not provide a satisfactory account of the process (i.e., the low SI for M_1_ with the Performance judgments).

The results further indicate that additive cue integration is more often associated with Intuition(B) while multiplicative cue integration can potentially be associated with both Analysis(B) and Intuition(B). In line with our hypotheses, we see that with easy access to a known declarative rule (in the Math, Area, Speed, and EV conditions) participants clearly relied on multiplicative integration based on Analysis(B). However, without such a rule and/or in more subjective domains (in the Performance and WTP condition) participants instead resorted to either Intuition(B) additive integration or Intuition(B) multiplicative integration as indeed suggested based on previous findings by, for example, Brehmer (1994), for the former type of integration, and Andersson (1996, 2008), for the latter. However, the marginal support for the additive model as well as the low fit for either model in the Performance condition makes it clear that this contingency between process and integration strategy should be interpreted with care and will need to be further replicated in other content domains.

We found no support for Brunswik’s claim for larger errors with Analysis(B) processes in Experiment 2. Both in Brunswik (1956) and in Experiment 1 the errors were smaller in the perceptual task than in the conceptual task. That this is in not confirmed for the symbolic tasks in Experiment 2 may indicate that the veracity of Brunswik’s claim regarding the relative size of errors is confined to the very well attuned Intuition(B) processes involved in perception and does not generalize to conceptual tasks. Conceptual tasks may be more affected by Analysis(B) processes and/or involve evolutionary more recent task requirements that draw on less fine-tuned Intuition(B) process, as compared to the fine-tuned perceptual judgments of size.

## Experiment 3: Quasi-rationality in multiple-cue judgments

Experiments 1 and 2 emphasized either perceptual tasks or tasks for which the content suggested a more or less obvious explicit algorithm to be mentally “crunched” (e.g., for area). However, much of the discussion of “intuition and analysis” in the Brunswikian tradition has involved research on multiple-cue judgment, where participants discover the task structure from prolonged training with outcome feedback (see Hammond & Stewart, 2001). In multiple-cue judgment tasks, the cue integration can, in principle, involve two different sorts of rule-based processes: (i) the “crunching” of numerical cues according to an explicit formula, and (ii) the “quasi-rational,” informal assessment and integration of the effect of the cues on the criterion.

The purpose of Experiment 3 was to validate this quasi-rational interpretation of the observed good fit of linear regression models in Brunswikian research on multiple-cue judgment (Brehmer, 1994; Karelaia & Hogarth, 2008; Juslin et al., 2008). In this view, people have rule-like beliefs about how each cue relates to the criterion (e.g., that fever is a cue for pneumonia), but the cues are not integrated by any explicit symbolic formula, but informally by taking stock of the likely, approximate impact of each cue on the criterion. This quasi-rational mix of rule-like knowledge and informal cue integration is often used to explain the inconsistency observed in 50 years of lens-model research (Karelaia & Hogarth, 2008). Although the processes are “analytic” in that people abstract rule-like beliefs, the quasi-rational cue integration predicts that these cue abstraction processes should disclose the empirical hallmarks of Intuition(B).^7^ The process is noisy and inconsistent from judgment trial to judgment trial and gravitates to the default of linear additive integration of the cues (Brehmer, 1994; Karelaia & Hogarth, 2008; Juslin et al., 2008).

In Experiment 3, we applied the PNP model to classical multiple-cue judgments. Considering the vast amounts of data already published on multiple-cue judgment (Brehmer, 1994; Karelaia & Hogarth, 2008), we decided to apply the PNP model to an already published data set that was easily available to us (Karlsson et al., 2007), which also mapped conveniently onto the simulations presented in Table 1, above (see the section on Model Recovery, above). Experiment 1 in Karlsson et al. (2007) involves a multiple-cue judgment task where participants learn from outcome feedback training to use four continuous cues to predict a continuous criterion, either in a task where the cues combine by additive integration (Equation 5) or by nonadditive integration, given by

11 $$ \hat{y}=509.05+.54{e}^{\left(4{x}_1+3{x}_2+2{x}_3+1{x}_4\right)/18}, $$

with the constants chosen to define the same training criterion range in both conditions.

The hypothesis tested in Karlsson et al. (2007) was that the additive task should invite rule-based additive cue abstraction, and the nonadditive task should invite exemplar-based strategies (i.e., the generalized context model; Nosofsky, 2011, but applied to a continuous criterion; see Karlsson et al., 2007). Previous research suggests that people are spontaneously inclined to engage in additive integration of externally provided cues, much as if they implemented a multiple linear regression model with the cues as independent variables, but which only contains main effects and no interaction terms (Brehmer, 1994; Karelaia & Hogarth, 2008). Therefore, people often have difficulty mastering nonadditive tasks by cue abstraction strategies (Juslin et al., 2008; Juslin et al., 2009). In lieu of access to any obvious explicit integration rules that can be the subject of Analysis(B) (as for several tasks in Experiment 2, like Area), in tasks with feedback learning, they often shift to exemplar-based memory when tasks require multiplicative cue integration (see also Hoffmann et al., 2014, 2016; Juslin et al., 2008; Juslin et al., 2009; Juslin et al., 2003; Karlsson et al., 2007; Little & McDaniel, 2015; Pachur & Olsson, 2012; Platzer & Bröder, 2013; von Helversen et al., 2010; von Helversen & Rieskamp, 2009, for similar results). The general context model entails judging the criterion of a task based on informal similarity-based weighting of previous exemplars (Nosofsky, 2011). Participants relying on exemplar-based memory are thus expected to be Intuitive(B). However, if people instead judge the criterion based on root-memorization of previous exemplars, their responses should instead show the hallmarks of Analysis(B).

The participants trained with outcome feedback for 300 trials and thereafter received a test phase with 44 trials (without outcome feedback), which also required extrapolation beyond the training range to investigate whether the participants primarily relied on rule-based or exemplar-based strategies. Both the measures of extrapolation and the computational modeling supported the hypothesis that the participants in the additive task primarily engaged rule-based cue-abstraction, while the participants in the nonadditive task primarily engaged exemplar-based strategies.^8^

The modeling in Karlsson et al. (2007) was based on the standard assumption of a homogeneous Gaussian noise. With the PNP model, we can now test the assumption of quasi-rationality often made in Brunswikian research on multiple-cue judgment: The beliefs that people have abstracted about how the cues are related to the criterion are integrated by an informal and inconsistent process with the empirical hallmarks of Intuition(B). Note that whereas the ability to extrapolate or not identifies if the beliefs involve rule-based and generalizable knowledge or exemplar-memory based knowledge, the PNP model identifies whether the process discloses the systematicity typical of Analysis(B) or the noisiness of typical Intuition(B). The quasi-rationality should imply both rule-based generalizability and the noisiness of Intuition(B) processes.

### Method

The PNP model was applied to the data set from Karlsson et al. (2007), with an additive model (see Equation 5) and an exemplar-based model on the equation

12 $$ y=\frac{\sum_{j=1}^{300}\exp \left(-\delta {\sum}_{i=1}^4{\omega}_i\left|{x}_i-{x}_{ji}^{\ast}\right|\ \right)\times {c}_j}{\sum_{j=1}^{300}\exp \left(-\delta {\sum}_{i=1}^4{\omega}_i\left|{x}_i-{x}_{ji}^{\ast}\right|\ \right)}, $$

where *y* represents a weighted average of the criteria *c*_*j*_ of each of the 300 exemplars from the feedback training based on the similarity of the exemplars x^*^_j1_ … x^*^_j4_ to the probe *x*_1_ … *x*_4_; *δ* is a sensitivity parameter representing discriminability in psychological space and *ω*_*i*_ are weight parameters representing attentional weights for each cue variable. Thus, the design of the analysis was similar to Experiment 2, in the sense that two different cue-integration rules (additive vs. exemplar-based) were explored, each of which could potentially be realized as an Analysis(B) or an Intuition(B) type process. As before, the cue-integration rule was determined by the best fitting model, while the contribution of Analysis(B) or Intuition(B) was signaled by the value of the *λ* parameter for the best fitting model.

### Results and discussion

The *λ* parameters for the best-fitting models were high for all participants (median = .977, min = .854, max = .996). Accordingly, K-S tests could refute normality for 0% of participants in the additive condition and for 12.5% in the nonadditive condition. This indicates that participants predominantly used Intuition(B) processes, most likely on account of the probabilistic feedback format (which makes inductively inferring the integration rule and the exact objective cue weights very difficult) and the complexity of the nonadditive task. Median adjusted *R*^2^ for the best models in the two conditions were Adj. *R*^2^ = .723 for the additive condition and Adj. *R*^2^ = .529 for the nonadditive condition.^9^ The proportions of best fitted models mostly match those presented in Karlsson et al. (2007), with 56.3% cue abstraction model in the additive condition (81% in the original study) and 62.5% exemplar-based model in the nonadditive condition (62.5% in the original study).^10^

#### Conclusions

The best-fitting version of the cue abstraction model in the additive task confirms the quasi-rational nature of the process: we observe both the generalizability of a rule-based strategy and the noisiness that is the empirical hallmark of an Intuition(B) process. By combining an experimental design requiring rule-based extrapolation with application of the PNP model, we can confirm the interpretation that people engage in a noisy quasi-rational cue integration process in multiple cue judgment (Hammond & Stewart, 2001).

In the nonadditive task, due to the complex integration rule in conjunction with the probabilistic feedback, the participants instead had to engage an Intuition(B) exemplar-based memory strategy, or, for a minority of participants, approximate the task with Intuition(B) additive integration strategy. This is in comparison to Experiment 2 where the PNP modeling of a cue integration task with a simpler multiplicative rule yield the empirical signs of Analysis(B). Thus, both in Experiments 2 and 3 we observed departures from the default of additive cue integration often observed in multiple-cue judgments, but the route of departure depends on the circumstances. In Experiment 2, the nonadditive cue integration could be addressed by Analysis(B) processes to the extent that the task invited salient explicit equations to execute (e.g., for area), while in Experiment 3, the content of the nonadditive multiple-cue judgment task affords no salient such explicit rule and the participants instead had to turn to the use of exemplar memory. The PNP model can thus successfully identify to what extent participants engage in Analysis(B) or Intuition(B) cue integration in different cue-integration tasks, where participants are hypothesized to engage different processes depending on, for example, the complexity of the normative integration rule and/or the access to known declarative integration rules.

## General discussion

The aim of the present study was to reintroduce the intriguing operational definition of intuition and analysis proposed by Egon Brunswik (1956), and to explore how these ideas can be developed into a computational model that can stimulate novel insights and hypotheses about human cognition. One original implication by this proposal is to take the errors in judgment not only or primarily as a nuisance in the scientific process but also as a positive tool to understand the nature of the process. By this method, two distinct kinds of cognitive processes can be successfully identified by purely empirical properties of the judgment distributions.

Brunswik argued that Intuition(B) is approximate and robust in nature, giving rise to a small Gaussian error that generally does not lead the agent too far astray. These processes rely on error-perturbed (noisy) representations from perceptual coding or readings of internal states (e.g., preferences). Intuition(B) processes also involve cue-integration processes by which often vague beliefs about relations (e.g., smoking cause cancer) are translated, online and ad hoc, into quantitative judgments by an inconsistent process that produces different judgments from time to time for the same stimuli—similar to the inconsistency and “lack of cognitive control” typically observed in multiple-cue judgments. Analysis(B), on the other hand, is deterministic and is often precisely correct, but characterized by occasional large errors. These processes rely on error-free representations deriving from explicit rules that (for the most part) allow exact outputs.

To apply, explore, and extend Brunswik’s ideas, we proposed the precise/not precise (PNP) model, which uses a mixture distribution to distinguish between precise and nonprecise responses relative to a predefined computational-level cognitive algorithm. Thus, the model samples from two different distributions: error-free application of the cognitive algorithm, and responses that are affected by errors in execution of the algorithm. In contrast to Brunswik’s (1956) original formulation, however, these error distributions are not assumed to be distributed around the correct criterion values, but can refer to the output of any cognitive algorithm. Model recovery demonstrated that when the generative model for the data is known, the PNP correctly recovers the underlying process, and often with much more accurate parameter estimates.

Experiment 1 demonstrated that the PNP model can recover Intuition(B) processes in perceptual tasks and Analysis(B) processes in conceptual tasks in the same context that originally motivated Brunswik’s claim for different error distributions. More specifically, we replicated the claims about the differential shapes of the error distributions in the two tasks and demonstrated that the PNP model appropriately identifies the two processes based on these error distributions. The results also confirmed Brunswik’s claim that Analysis(B) processes are associated with larger, although less frequent, errors than the perceptual Intuition(B) processes.

Experiment 2 showed that the error distributions that define Intuition(B) processes are present and identifiable by the PNP model also in purely symbolic and conceptual tasks involving no perceptual noise. That conceptual tasks should involve intuitive thought-processes in some sense is not surprising, of course. But to our knowledge, this is the first empirical demonstration showing that cognitive-modeling efforts can be construed so that intuitive and analytical thought processes can be identified specifically by the shape of the error distributions. In contrast to Experiment 1, there was no strong evidence that the magnitude of errors differed between processes. It is possible that perceptual processes are more “fine-tuned” as a result of hard-wired processes shaped by evolution, a fine tuning that has yet to materialize for evolutionary relatively new processes The results also demonstrate that the PNP model often recovers the parameters of the process more effectively than a standard regression model with homogeneous Gaussian error, suggesting that the standard Gaussian assumption is often an incorrect specification of the error distribution (e.g., consider the examples of ID = 24 in Fig. 1 and the results for Experiment 2).

Experiment 3 verified the quasi-rational character of the cue integration in symbolic multiple-cue judgments, as often assumed but not, in any direct way, tested. In the additive task, the cognitive process was, on the one hand, rule-based, allowing extrapolation beyond the training range, and, on the other hand, noisy and inconsistent, as captured by an Intuition(B) process. The process is accordingly different in nature from the rule-based Analysis(B) processes observed in Experiment 2, which entails “number crunching” in accordance with a declarative rule.

Together, these results validate and extend Brunswik’s claims concerning Intuition(B) and Analysis(B). In the remainder of this article, we turn to a discussion of the wider theoretical and methodological implications of Brunswik’s claims and the PNP framework, as well as to discussing the limitations, and highlighting promising venues for future research.

### Brunswik’s definition and the dual-system definitions

The distinction between intuition and analysis in dual-systems theories (e.g., Evans, & Stanovich, 2013; Kahneman & Frederick, 2002; Sloman, 1996) pervades research in numerous areas (e.g., Ashby & Valentin, 2017; Juslin et al., 2008; Pacini & Epstein, 1999; Sundh & Juslin, 2018; von Helversen & Rieskamp, 2009). Although it has proven difficult to identify properties common to all dual-systems theories (Evans, 2008), it is typically assumed that analytic processes are slow, rule-based, and explicit processes, drawing on deliberate and controlled thought in working memory. Intuitive processes are faster, based on similarity, and less constrained by working memory capacity (see, e.g., Evans, 2008, for a review).

Although this literature seems to capture a popular appreciation, it has been repeatedly criticized (Gigerenzer, 2010; Keren & Schul, 2009; Kruglanski & Gigerenzer, 2011; Melnikoff & Bargh, 2018) for confusing distinctions, like *implicit* versus *explicit*, *automatic* versus *controlled* processes, *impulsiveness* versus *reflection*, and *intuition* versus *analysis*. Identifying which process is used has also proven challenging (Dane & Pratt, 2009; Hammond et al., 1983): Inspection times and response times have been proposed as independent criteria (see Osman, 2004; Pashler, 1994), but seem to have limited validity, at times confusing the two processes (Furlan, Agnoli, & Reyna, 2016; Krajbich, Bartling, Hare, & Fehr, 2015).^11^ Brunswik’s claim offers a complementary strategy that defines the processes in terms of properties of empirical data that may be a useful alternative to the use of vague and verbal concepts (e.g., automatic vs. controlled; impulsive vs. reflective; explicit vs. implicit). At present, we regard as empirical questions how the cognitive properties captured by Analysis(B) and Intuition(B) processes relate to the many distinctions proposed in the existing dual-systems literature (Evans, 2008). In the following, we nonetheless provide a tentative discussion of when we expect that the Brunswikian and the dual-system accounts are more likely to converge, and when they are less likely to do so.^12^

#### Similarities

One of the most influential discussions of intuition and analysis has been in terms of “attribute substitution” (Kahneman & Frederick, 2005), where the assessment of a normative and hard to assess variable (e.g., probability) is replaced by a simpler and intuitive “natural assessment” (e.g., representativeness). The use of such heuristic intuitive variables often comes at the price of violating certain normative (analytic) principles, leading to biases.

The heuristics and biases program indeed also has links to Brunswik. As noted by Kahneman and Frederick (2005), the original formulation of the program “was intended to extend Brunswik’s (1943) analysis of the perception of distance to the domain of intuitive thinking” (p. 268). Not surprisingly then, natural assessments that include physical properties such as size and distance and more abstract properties such as similarity, cognitive fluency, and affective valence (Kahneman & Frederick, 2005) will presumably be defined as intuitive also in the Brunswikian perspective—that is, what we refer to as Intuition(B). The intuitive assessments of representativeness that have been used to demonstrate, for example, base-rate neglect or the conjunction fallacy are therefore perfectly consistent with the definition of Intuition(B) suggested by Brunswik’s distinction. Consider, for example, the classic Linda problem, used to illustrate the use of the representativeness heuristic:

Linda is 31 years old, single, outspoken, and very bright. She majored in philosophy. As a student, she was deeply concerned with issues of discrimination and social justice and also participated in antinuclear demonstrations. Which of the following is more likely?

(A) Linda is a bank teller.

(B) Linda is a bank teller and is active in the feminist movement.

The conjunction error is the assessment that the conjunction, *bank teller and feminist*, is more likely than one of its constituents, *bank teller*, presumably because Linda is perceived to be particularly representative of the category “feminists” (Tversky & Kahneman, 1983; see also Costello & Watts, 2014; Nilsson, Juslin, & Winman, 2016). The similarity-based impression that Linda “must be” a feminist is indeed an example of an intuition that is also likely to surface in analysis using the PNP model.^13^ A difference is that with the PNP model, this issue need not be the subject of speculation; it is solved empirically by applying the model to the data.

#### Differences

Other demonstrations of variable substitution in the heuristics-and-biases literature, especially those connected with the cognitive reflection test (CRT), are, however, likely to be interpreted differently in terms of the PNP model. Consider the following question: “A bat and a ball cost $1.10 in total. The bat costs $1 more than the ball. How much does the ball cost?” Most people guess “10 cents,” presumably because “$1 + 10 cents = $1.10.” The PNP model is likely to classify this not as the result of an intuitive variable substituting for an analytic variable (Kahneman & Frederick, 2005), but as execution of the wrong analytic algorithm (i.e., otherwise most people would not produce exactly the same answer). In the context of the PNP model, the CRT is not a test of intuition or analysis, but on the ability to critically reflect on the output of the process (as indeed suggested by the name CRT). As with Linda, people get this problem wrong, but not primarily because of an intuition that it “must be” the correct answer, but rather due to the mindless execution of an analytic algorithm that is strongly invited by the numbers.

A major difference from the dual-systems framework, however, is that there is nothing in the Brunswikian framework suggesting that the Analysis(B) has priority, with the role of supervising and “censoring” the output of Intuition(B). Rather the two processes are likely to “supervise” each other. In the Linda problem, it may well be that an analytic rule from probability theory is suddenly retrieved and serves to overrule the strong intuition that there must be a higher probability that Linda is a feminist and a bank teller, than that she is a bank teller. But it might be just as common that diligent analytic solutions are supervised by and overruled by Intuitive(B) processes, as, for example, when a student performing a math problem that concerns computation of a person’s body weight, coming up with the answer of “5,000 kg,” will quickly appreciate that the analytic computation is wrong.

The distinction captured by the PNP model is not identical to the distinction between controlled and automatic processes (Atkinson & Shiffrin, 1968; Schneider & Shiffrin, 1977). Both the analytic crunching of numbers in mental algebra tasks (Analysis(B)) and the intuitive linear additive integration of cues in multiple-cue judgment tasks (Intuition(B)) are likely to involve controlled processes, and one reason for the inconsistency of the cue integration in multiple-cue judgment is probably the working-memory constraints on these processes. However, as we have seen, automatic perceptual and memory retrieval processes are likely to disclose the Intuition(B) properties. The explicit symbolic nature of the representations in Analysis(B) processes easily lend themselves to succinct verbal expression. Such verbal expressions of representations will only be available for some Intuition(B) processes, such as when applying a deterministic rule to noisy input (i.e., calculating the area of perceptual triangles in Experiment 1) or as when people are able to articulate the direction of cue–criterion relationship in multiple-cue judgment tasks, even though they cannot verbally express the integration rule. The issue of what aspects of the process that are “unconscious” in any more profound sense, as compared with just difficult to map onto explicit verbal representations, is more subtle. As already noted, the PNP distinction differs from the distinction between impulsiveness versus reflection, because both Intuition(B) and Analysis(B) processes can be used in a more or less reflective way.

### The PNP model as an alternative to traditional statistical modeling tools

Error distributions have been addressed in computational modeling in other domains, like, for example, visual short-term memory (Bays, Catalao, & Husain, 2009; Luck & Zhang, 2008; Ma, Husain, & Bays, 2014; Van den Berg, Shin, Chou, George, & Ma, 2012; Wilken & Ma, 2004), categorization learning (e.g., Ashby, Maddox, & Bohil, 2002), and choice problems (Birnbaum & Quispe-Torreblanca, 2018; Lee, 2018). In contrast to these previous approaches, the PNP model combines modeling with the rationale derived from Brunswik’s (1956) distinction between error distributions. The PNP model therefore raises new methodological issues.

As we have seen, a standard model with Gaussian error may sometimes erroneously specify the process, leading to false conclusions (e.g., the different conclusions suggested by the analyses for the participant in Fig. 1). In tasks with Analysis(B) processes, the PNP model allows the unpacking of the traditional notion of error into two components: the probability than an error occurs in the first place (*λ*), and the magnitude of the error in those cases where an error has occurred (*σ*). Thus, tasks that are solved by Analysis(B) can differ profoundly in their difficulty in terms of *λ* quite regardless of the magnitude of the errors when they do occur. Conversely, a task may be easy to perform decently, if the magnitude of the error is low (*σ*) also if errors are frequent (see Groß & Pachur, 2019, for a similar distinction in reconstructive memory).

On a more general note, the approach suggested by the PNP model could also be a principled and more general solution to the problem of how to treat outliers in the data analysis. The current practice is often to delete participants that are, for example, four or more standard deviations from the mean of the participants distribution. Potentially, the same problem could be addressed in formal modeling by allowing for heterogeneous error distributions in the models. After all, the application of the PNP model could be described as a matter of correctly identifying the correct parameters in the presence of severe outlier responses (see, e.g., Fig. 1).

### Limitations and future directions

There are, of course, limitations to the PNP model, some of which have already been addressed in this study. When the response variable is discrete with few response categories there is risk that the estimation of λ and σ become biased. There are, at least, two different ways to address this problem. As detailed in Appendix A, a first way is to view it is as a methodological problem, the solution to which is to use more refined methods to estimate the parameters. However, a different way to address the problem is conceptually, by acknowledging that the causes of this problem sometimes revolve around intrusion of constraints that indeed make the process more analytic and deterministic in the context of the PNP model. For example, the rounding of the responses to even digits in our (cultural) number system or preknowledge of a few admissible response alternatives, are constraints that make the responses more predictable by application of rule-like beliefs, reducing the error that is well captured by a Gaussian noise.

Another potential issue is that, if the correct algorithm is unknown, the PNP model might mistakenly categorize a process as Intuition(B) because the systematic variation produced by an unknown algorithm is modeled as noise. Although this sort of theory-dependence is difficult to completely eliminate in any cognitive modeling, it does imply a risk of bias when applying the PNP model to data. There are ways to mitigate this risk. First, it is imperative to make sure that the chosen model captures most of the systematic variation in the data—for example, by measures such as the saturation index (SI) used in this paper (see Experiments 1 & 2). Second, it is important to watch for regularities in the data that are not captured by the modeling. For example, in probability elicitation tasks, people sometimes prefer numbers divisible by 5 or 10, which might necessitate adding a rounding process to the model. Third, one should be aware that the Intuition(B) model is the “default,” in the sense that a participant will generally consider Intuition(B) if no Analysis(B) model is found, and interpret the data accordingly.

Although we have used Gaussian distributions in this study, and we believe that this represents a useful standard that can be applied to a great variety of data, it is possible that other types of distributions could be more suitable in certain cases. For example, evidence suggests that numerical estimates that cover large differences in magnitude might be better represented by a log-normal distribution (Dehaene, 2003; Feigenson, Dehaene, & Spelke, 2004). This would be a relatively simple alternative application of the PNP model, but it would necessitate theoretical assumptions regarding in which contexts noise are likely to be log-normally distributed. Because errors in Analysis(B) and Intuition(B) processes (as defined by us) spring from different sources, it is not necessarily the case that they share the same distribution, and it is possible that, for example, errors are log-normally distributed with Intuition(B), but not with Analysis(B).

While our results generally indicated support for the two distinct categories of Intuition(B) and Analysis(B), it is not inconceivable that other tasks, in other contexts, might result in a less bimodal distribution of *λ*. It is also possible that certain more complex tasks could induce systematic shifts between the two. This is no doubt a promising area for further study, and one where the PNP model, or extensions thereof, could prove useful.

Finally, we acknowledge that although we have tried to nurture one legacy after Brunswik (1956), the distinction between Analysis(B) and Intuition(B) in terms of the error distributions, we have not even started to address the other important legacy: to better understand how these different processes are selected by, and fit with the structure of, natural environments. The work on cognitive continuum theory (Hammond, 1996; Hammond et al., 1983) remains the most systematic attempt to address the functional role of analytic and intuitive cognitive processes, and we hope that the PNP model can be an additional tool in this important pursuit. A relevant avenue for future research is to investigate the prevalence of Analytic(B) and Intuitive(B) processes in environments with varying degrees of noise. A reasonable hypothesis is that Analytic(B) processes should be prevalent in deterministic environments, but Intuitive(B) processes become more and more prevalent with increasing degrees of probabilism. The main reason being that participants will experience that their Analytic(B) process is not providing them with correct predictions, and thus they might abandon it for an Intuitive(B) process.

### Conclusions

In this article, we have pursued Brunswik’s definitions of Intuition(B), defined by ubiquitous deviations from the algorithm, as described by the sampling from a *homogeneous* Gaussian error distribution, and Analysis(B), defined by a *heterogeneous* error distribution. We propose that Brunswik’s original insight, that the nature of the judgment error can be diagnostic of the nature of the underlying cognitive processes, is an underused approach that may prove useful both in the current debates on dual-systems theories and in other domains.

## Electronic supplementary material

ESM 1 (DOCX 33.4 kb)

ESM 2 (CSV 66 kb)

ESM 3 (CSV 131 kb)

ESM 4 (M 1 kb)

ESM 5 (M 1 kb)

## Footnotes

1 The Dirac delta function models the probability density over an idealized point mass as infinity, which renders maximum likelihood estimates meaningless.

2 The residuals in Fig. 1 suggests not only a spike at 0, but also a skewed asymmetric error variance. It is therefore interesting that already with the current error specification, the model is able to correctly identify the processes parameters (α, β), implying that 78% of the response are an exact, error-free calculation of the EV.

3 In Experiment 3 (reported below) we apply the PNP model to empirical data of the same type as simulated in the model recovery.

4 When some participant responses lack error variance, as in the current data set where people sometimes multiply flawlessly, it is recommended to put a lower bound on the error variance estimate, just above the value of *τ*. By using a lower bound we avoid a divisor of zero in the likelihood function, and by setting that lower bound above *τ,* we ensure that such participants are not mistakenly categorized as intuitive rather than analytic with respect to the maximum likelihood estimate. This will unavoidably introduce a bias in the error variance estimate, but because this bias is very small, and because these particular participants will be easy to distinguish, this is a minor problem.

5 The response variable was varied within subjects for the purposes of another project (focused on investigating directional sensitivity in task performance and the potential effects of automatized reasoning schemes) and will not be further analyzed and systematically addressed in this article.

6 The adjusted *R*^2^ for M_1_ in the Performance condition might seem surprisingly low, even for a badly fitting model, but it should be noted that adjusted *R*^2^ punishes low values of *R*^2^ more than high values. So, for example, for M_1_ in the math condition, median *R*^2^ = .961 and median adjusted *R*^2^ = .955, while for M_1_ in the performance condition, median *R*^2^ = .179 and median adjusted *R*^2^ = .021. Indeed, for especially badly fitted models, adjusted *R*^2^ can even result in negative values.

7 In previous research, these cue abstraction models have often been juxtaposed against exemplar-based models (e.g., Juslin et al., 2003), and, in comparison to exemplar-based models, cue abstraction models are “analytic” in the sense that they assume that people abstract explicit rules connecting the cues with the criterion. Nonetheless, the cue integration is not based on analytic crunching of the cue numbers according to a symbolic formula, but this integration occurs intuitively and informally by a sequential updating of an estimate of the criterion. For example, in Juslin et al. (2008), it is explicitly stated (on p. 264) that cue integration rule is “procedural,” in contrast to the cue–criterion relations that are part of the working memory content and which provide input to these processes.

8 A rule-based multiplicative cue abstraction model assuming cue integration according to Equation 11 was also fitted to the data in Karlsson et al. (2007) but this model was not supported by the results.

9 Because the experiment in Karlsson et al. (2007) contained no replication, we were unable to compute the SI.

10 Because all participants had very high values on *λ*, it was not possible to analyze correlation between *λ* and error magnitude.

11 The data in Experiments 1 and 2 were never collected with the instructions and response settings required for valid measurement of response times. Nonetheless, for the interested reader, we note that Bayesian Kendal’s tau indicated no correlation between mean response times and estimated *λ* in Experiment 1 (*r*_*τ*_ = −.077, BF_10_ = .253) and no correlations in Experiment 2 (M_1_: *r*_*τ*_ = .033, BF_10_ = .052; M_2_, *r*_*τ*_ = .038, BF_10_ = .158; Prod, *r*_*τ*_ = .080, BF_10_ = .256).

12 Due to the vastness of the dual-systems literature, we have chosen a select number of clear-cut comparisons—future research will have to expand this discussion further by incorporating also other conceptualizations (e.g., Mukherjee, 2010; Tenenbaum & Griffiths, 2001).

13 Note that the fact that people rely on representativeness or similarity to make the assessments in this task—and there indeed seems to be little alternative to use this impression in this specific task—need not imply that this is the cause of the conjunction fallacy. The conjunction fallacy is prevalent also in many situations where people cannot rely on representativeness, and the more fundamental underlying cause seems to be that people combine their probability assessments for the constituent events (however they are initially produced) by additive integration rather than the multiplicative integration rule implied by probability theory (see Juslin et al., 2009, for a discussion).
