# Supplementary material for: Precise/not precise (PNP): A Brunswikian model that uses judgment error distributions to identify cognitive processes
Source: Psychon Bull Rev. 2020 Sep 28;28(2):351–73. doi: 10.3758/s13423-020-01805-9 (PMC8062428; doi:10.3758/s13423-020-01805-9)
Supplement: Supplementary file 1 — (DOCX 33.4 kb) [file 13423_2020_1805_MOESM1_ESM.docx]

**Appendix A:**

**Correction for a Limited Response Scale When Applying the PNP Model**

When the data are discrete rather than continuous, and when the range of the response variable is severely limited, one runs into the risk that participants make precise responses “by chance.” For example, one might envision a (somewhat extreme) situation in which the response variable of a cue integration task can only take on integer values between 1 and 5. In such a case, even the participants who use a distinctly intuitive strategy will no doubt occasionally produce responses which correspond to the exact function value and which, consequently, are treated as precise in the context of the PNP model. There are two ways to circumvent this problem.

The simplest (and in our opinion the best) solution is to specify the values of *λ* before applying the model to data, corresponding to the proportions of precise and non-precise responses one expects in the processes one wishes to model. In those particular cases where one expects precise responses to occur during intuitive processes, one simply takes this into consideration when choosing the values of *λ*. For example, if one expects approximately, say, a fifth of all responses made during an intuitive process to correspond with the exact function value by chance, one specifies the intuitive model(s) to include a value of *λ* = .80. The value of *λ* included in the analytic model(s) will likewise be specified according to the amount of errors one expects to occur during an analytic process. This may or may not match the inverse of the *λ* used for the intuitive process, depending on what the hypothesized processes entail.

An alternative solution, which can be used if one is wants to estimate *λ* rather than specifying its value before modeling, is to correct this estimation by an adjustment based on the probability that values drawn from the non-precise distribution falls into a specified range within where they will be treated as precise (given by the cumulative distribution function). For example, if the response variable only takes on integer values, one can use the cumulative distribution function to estimate the probability that non-precise values fall within the range [-0.5, 0.5]. This adjusted likelihood function, in which ±*q* defines the region in which non-precise responses might be mistaken as precise and *F* is the cumulative distribution function, is defined by the following equation:

|  | $f_{A}\left(\left.y\right\vert\lambda,g,\mathbf{x},\mathbf{\theta},\sigma,\tau\right)=\lambda\left(f\left(\left.y\right\vert g,\mathbf{x},\mathbf{\theta},\sigma\right)+A\right)+\left(1-\lambda\right)\left(f\left(\left.y\right\vert g,\mathbf{x},\mathbf{\theta},\tau\right)-A\right)$   | (A1) |
| --- | --- | --- |

This adjustment should be applied with care, for two primary reasons: First, it adds a significant degree of complexity to the model. Second, this presupposes a cognitive mechanism in which noise is routinely “rounded off” during the judgment process, which may not necessarily be representative of reality, depending on the structure of the task and the nature of the function one wishes to evaluate.

**Appendix B:**

**Task Instructions Experiment 1**

**Conceptual Triangle**

In this experiment you will estimate the area of 50 right-angled triangles, presented one by one. Such a triangle could look like the triangle below, where the blue quadrant shows the right angle.

You will be presented with information about the length of the base and the height of the triangles you will assess. You will not be shown a picture of the triangle. Your task is to estimate the area of the triangles in square centimeters (cm^2^). Answer with an integer or with up to one decimal. Use decimal point as decimal separator if you answer with a decimal.

**Perceptual Triangle**

In this experiment you will estimate the area of 50 right-angled triangles, presented one by one. Such a triangle could look like the triangle below, where the blue quadrant shows the right angle.

You will be presented with pictures of the triangles you will assess. Your task is to estimate the area of the triangles in square centimeters (cm^2^). Answer with an integer or with up to one decimal. Use decimal point as decimal separator if you answer with a decimal.

**Perceptual Blob**

In this experiment you will estimate the area of 50 irregularly shaped figures, presented one by one. Such a figure could look like the figure below.

You will be presented with pictures of the irregularly shaped figures you will assess. Your task is to estimate the area of the figures in square centimeters (cm^2^). Answer with an integer or with up to one decimal. Use decimal point as decimal separator if you answer with a decimal.

**Appendix C:**

**Descriptive Statistics Experiment 1**

This Appendix includes the descriptive statistics for (*i*) participant’s area estimates in the experimental task, (*ii*) correlation coefficients *r* between response and criterion, (*iii*), RMSE from an error-free calculation of triangle area, and (*iv*) the response times (RT). All information is summarized in Table C1.

Table C1.

*Inferred Values by Participants in the Experimental Task for Each Condition and Response Variable (Medians along with 25^th^ and 75^th^ Quartiles).*

|  | Measure | | | | |
| --- | --- | --- | --- | --- | --- |
| Condition | | Estimate | *r* | RMSE | RT |
| Triangle (conceptual) | | 17.5 (17.5; 17.5) | .979 (.808; .996) | 1.27 (.566; .5.59) | 6.08 (3.08; 12.3) |
| Triangle (perceptual) | | 15.0 (14.0; 17.5) | .866 (.743; .904) | 4.56 (4.08; 7.39) | 9.94 (5.13; 15.1) |
| Blob (perceptual) | | 9.25 (6.00; 17.0) | .782 (.701; .894) | 8.59 (6.67; 12.5) | 4.16 (3.31; 4.91) |

**Appendix D:**

**Task Instructions Experiment 2**

*Abstract mathematics.* Your task is to solve the following math problems correctly as fast as possible. The numbers involved may involve either proportions (e.g., .50) or integers (e.g., 50).

Two numbers are .20 and 20. What is the product of these numbers?

______

The product of two numbers is 4. One number is 20. What is the other number? ______

The product of two numbers is 4. One number is .20. What is the other number? ______

*Area of a plank.* Your task is to estimate the following lengths and areas correctly as fast as possible. The numbers involved may involve either proportions (e.g., .50) or integers (e.g., 50).

The width of a plank is .20 meters and its length is 20 meters. What area in m^2^ is covered by the plank? ______

The area covered by a plank is 4 m^2^. The length of the plank is 20 meters. What is the width of the plank? ______

The area covered by a plank is 4 m^2^. The width of the plank is .20 meters. What is the length of the plank? ______

*Objective expected values.* Your task is to estimate the following components of a lottery correctly as fast as possible. Each lottery is on the form that there is a given probability to win a positive sum of money and if you do not win you receive nothing (e.g., a .50 probability to win 100 SEK, otherwise your receive 0 SEK).

The probability to win is .20 and if you win you receive 20 SEK (and 0 SEK otherwise): What is the long run average gain if you repeatedly partake in this lottery? ______

The long run average gain if you repeatedly partake in a lottery is 4 SEK. The amount you can win each time you partake in the lottery is 20 SEK (and you receive 0 SEK if you do not win). What is the probability of winning each time you partake in the lottery? ______

The long run average gain if you repeatedly partake in a lottery is 4 SEK. The probability that you win each time you partake in the lottery is .20. What is the amount you can win each time in the lottery (assuming that you get 0 SEK if you do not win)? ______

*Willingness to pay.* Your task is to estimate the subjective value you attach to components of a lottery as fast as possible. Each lottery is on the form that there is a given probability to win a positive sum of money and if you do not win you receive nothing (e.g., a .50 probability to win 100 SEK, otherwise your receive 0 SEK).

The probability to win is .20 and if you win you receive 20 SEK (and 0 SEK otherwise): What is the highest price you would be willing to pay to partake in this lottery? ______

The amount you win if you partake in the lottery is 20 SEK (and you receive 0 SEK if you do not win). What is the smallest probability of winning in this lottery that would make you prepared to partake in this lottery at a ticket cost of 4 SEK? ______

The probability that you win if you partake in the lottery is .20. What is the smallest winning money sum (assuming that you get 0 SEK if you do not win) that would make you prepared to partake in this lottery at a ticket cost of 4 SEK? ______

**Appendix E:**

**Descriptive Statistics Experiment 2**

This Appendix offer the descriptive statistics for (*i*) participant’s judgments in the experimental task (Table E1), (*ii*) correlation coefficients *r* between response and criterion (Table E2), (*iii*), RMSE from an analytical multiplicative integration model (Table E3), and (*iv*) the median response times (Table E4).

Table E1.

*Inferred Values by Participants in the Experimental Task for Each Condition and Response Variable (Medians along with 25^th^ and 75^th^ Quartiles).*

|  | Response Variable | | |
| --- | --- | --- | --- |
| Task Content | M_1_ | M_2_ | Product |
| Math | .500 (.500; .600) | 50.0 (50.0; 57.5) | 20.0 (20.0; 24.0) |
| Area | .500 (.500; .600) | 50.0 (40.0; 60.0) | 20.0 (18.0; 24.0) |
| Performance | .400 (.300; .500) | 35.0 (30.0; 40.0) | 32.0 (30.0; 33.5) |
| Speed | .500 (.460; .600) | 50.0 (40.0; 50.0) | 19.0 (16.0; 20.0) |
| EV | .500 (.400; .560) | 45.0 (40.0; 50.0) | 20.0 (20.0; 22.0) |
| WTP | .700 (.600; .775) | 60.0 (55.0; 70.0) | 15.0 (10.0; 20.0) |

Table E2.

*Correlation Coefficients (r) for Each Condition and Response Variable (Medians along with 25^th^ and 75^th^ Quartiles).*

|  | Response Variable | | |
| --- | --- | --- | --- |
| Task Content | M_1_ | M_2_ | Product |
| Math | .980 (.255; 1.00) | .969 (.846; 1.00) | .999 (.871; 1.00) |
| Area | .972 (.330; .997) | .870 (.726; .974) | .965 (.705; .996) |
| Performance | .257 (.103; .773) | .730 (.604; .771) | .884 (.835; .914) |
| Speed | .938 (.256; .977) | .964 (.899; 1.00) | .983 (.811; .995) |
| EV | .815 (.110; .988) | .877 (.593; .997) | .781 (.600, .996) |
| WTP | .822 (.633; .895) | .744 (.539; .837) | .897 (.738; .957) |

Table E3.

*Root-Mean-Square Errors (RMSE) from an Analytical Multiplicative Integration Model for Each Condition and Response Variable (Medians along with 25^th^ and 75^th^ Quartiles).*

|  | Response Variable | | |
| --- | --- | --- | --- |
| Task Content | M_1_ | M_2_ | Product |
| Math | .790 (.000; 8.63) | 1.61 (.000; 6.05) | 2.28 (.000; 7.71) |
| Area | 4.76 (1.41; 13.6) | 2.12 (.000; 11.9) | 4.01 (1.19; 8.54) |
| Performance | 10.4 (9.92; 13.6) | 7.96 (6.87; 10.9) | 9.29 (7.56; 13.8) |
| Speed | 3.18 (1.74; 5.12) | 3.70 (2.55; 25.5) | 3.39 (.000; 5.09) |
| EV | 10.7 (.710; 16.6) | 4.91 (2.12; 20.9) | 5.83 (1.06; 11.9) |
| WTP | 13.9 (8.62; 17.3) | 11.2 (5.66; 14.8) | 16.7 (7.56; 35.6) |

Table E4.

*Response Times in Seconds for Each Condition and Response Variable (Medians along with 25^th^ and 75^th^ Quartiles).*

|  | Response Variable | | |
| --- | --- | --- | --- |
| Task Content | M_1_ | M_2_ | Product |
| Math | 14.2 (11.1; 18.9) | 13.1 (10.2; 20.1) | 12.0 (9.10; 17.3) |
| Area | 15.6 (11.5; 19.0) | 15.6 (11.0; 20.0) | 16.6 (12.0; 18.2) |
| Performance | 16.9 (13.6; 19.6) | 16.6 (14.3; 23.0) | 17.3 (15.5; 19.8) |
| Speed | 20.3 (9.50; 23.7) | 20.7 (15.4; 24.1) | 19.8 (13.0; 26.2) |
| EV | 21.2 (17.4; 24.0) | 26.4 (20.6; 30.2) | 20.9 (14.6; 26.7) |
| WTP | 18.7 (14.5; 21.3) | 20.3 (14.9; 23.6) | 14.8 (12.7; 17.2) |
